# Probing hair cell’s mechano-transduction using two-tone suppression measurements

**DOI:** 10.1038/s41598-019-41112-5

**Published:** 2019-03-15

**Authors:** Wenxiao Zhou, Jong-Hoon Nam

**Affiliations:** 1 Department of Mechanical Engineering, University of Rochester, Rochester, NY 14627 USA; 2 Department of Biomedical Engineering, University of Rochester, Rochester, NY 14627 USA

## Abstract

When two sound tones are delivered to the cochlea simultaneously, they interact with each other in a suppressive way, a phenomenon referred to as two-tone suppression (2TS). This nonlinear response is ascribed to the saturation of the outer hair cell’s mechano-transduction. Thus, 2TS can be used as a non-invasive probe to investigate the fundamental properties of cochlear mechano-transduction. We developed a nonlinear cochlear model in the time domain to interpret 2TS data. The multi-scale model incorporates cochlear fluid dynamics, organ of Corti (OoC) mechanics and outer hair cell electrophysiology. The model simulations of 2TS show that the threshold amplitudes and rates of low-side suppression are dependent on mechano-transduction properties. By comparing model responses to existing 2TS measurement data, we estimate intrinsic characteristics of mechano-transduction such as sensitivity and adaptation. For mechano-transduction sensitivity at the basal location (characteristic frequency of 17 kHz) at 0.06 nm^−1^, the simulation results agree with 2TS measurements of basilar membrane responses. This estimate is an order of magnitude higher than the values observed in experiments on isolated outer hair cells. The model also demonstrates how the outer hair cell’s adaptation alters the temporal pattern of 2TS by modulating mechano-electrical gain and phase.

## Introduction

The cochlea encodes acoustical stimulations into neural signals. The functional characteristics of hearing (tuning and amplification) are primarily determined at the mechanical level^1^. The cochlear cavity is divided into three fluid-filled spaces by two partitions. The basilar membrane is a mechanical partition between cochlear cavities (scala media and scala tympani)—it is a stiff plate-like structure embedded with collage fiber layers that vibrates due to differential fluid pressures between the two cavities. Most mechanical responses of the cochlea have been measured at the basilar membrane. Acoustic signals are carried in the form of traveling waves along the basilar membrane^2^. As the traveling waves propagate from the base towards the apex, the vibration amplitude of the basilar membrane increases, peaks at a location specific to the frequency and vanishes^3^. The responses of healthy cochlea are nonlinear so that the cochlea responds more sensitively to small sounds than loud sounds. This nonlinearity has been observed at the mechanical level: the vibration amplitude of the basilar membrane grows linearly with stimulus level at low intensities but grows more slowly at high intensities^4–6^.

**Figure 1:**
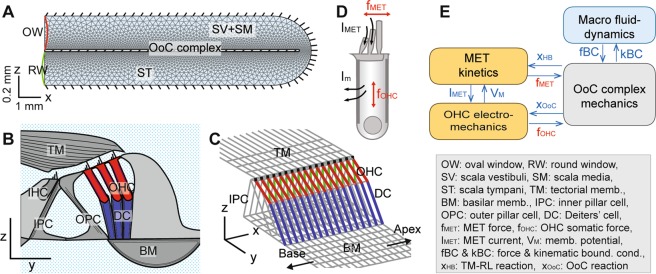
Virtual Cochlea—a computer model of cochlear MET. (**A**) Cochlear fluid dynamics: The cochlear duct is represented by a fluid-filled slender chamber divided into the top and the bottom fluid spaces by the elastic OoC complex. Fluid mesh is refined near the interacting surfaces. (**B**) Schematic drawing of the OoC complex. (**C**) 3-D FE model of the OoC with the realistic geometrical and mechanical properties of the gerbil cochlea. (**D**) Electro-mechanics of the OHC: including active forces from both gating of the MET channel in the hair bundle (*f*_*MET*_) and somatic motility (*f*_*OHC*_). (**E**) Relationship between Virtual Cochlear components (MET channel, OHC electro-mechanics, OoC complex micromechanics, and macro fluid dynamics).

The organ of Corti (OoC), the sensory epithelium of the cochlea, is sandwiched between two acellular matrices—the basilar and tectorial membranes (Fig. 1B). The OoC consists of two different types of receptor cells (inner and outer hair cells: IHC and OHC) and supporting cells. While the receptor cells transduces mechanical stimuli into neural impulses, the supporting cells play two roles: to form a mechanical scaffold and to maintain electro-chemical separation between scala media and scala tympani. Since von Békésy^7^, cochlear mechanics has long been based on basilar membrane mechanics including the measurements of Rhode and his colleagues^4,5^. Because of insufficient data, the OoC mechanics have been assumed kinematical^8–10^ (*i.e*., the OoC is considered as a rigid body, hence the displacement ratio between of OoC structures were assumed constants). Recent observations, however, reveal that the OoC is fully deformable, and that there exist different vibration patterns in OoC depending on active feedback of the OHCs^11–13^.

The receptor cells have developed microvilli protruding out of their apical surface called the stereocilia^14^. The mechano-electrical transduction (MET) channels are located at the tips of the stereocilia^15^. The tips of the tallest stereocilia row are attached to the tectorial membrane. The relative motion between the OoC and the tectorial membrane deflects the stereocilia to activate MET. The MET current modulates the receptor potential of the hair cell. In the case of the IHC, the receptor potential triggers the neural spikes of afferents. For the OHC, the primary functional consequence of its receptor potential is to generate mechanical force for cochlear amplification^16^. Cochlear nonlinearity depends on electro-mechanical feedback from the OHCs^17^.

According to the prevailing theory, cochlear nonlinearity originates from nonlinear MET of the OHC. Because the hair cell MET is central to cochlear function, great effort has been paid to quantify the biophysical properties of MET^18,19^. Hair cell MET has been extensively investigated at the cellular level through electrophysiological approaches. The MET sensitivity (*i.e*., MET current versus the hair bundle displacement relationship) is a rudimentary property that has been reported in many studies. The MET sensitivity varies from measurement to measurement by as much as an order of magnitude^20,21^. In various experiments, researchers have also observed the decay of MET current with sustained hair bundle deflection, a phenomenon which is called adaptation^22^. It is another characteristic of hair cell MET that has been investigated widely^23,24^. The adaptation time constant has been measured to be a fraction of a millisecond for rat OHCs^25^. The mechanism and function of hair cell adaptation are still debated^26–28^ and most of these measurements were performed *in vitro* (with isolated cochlear tissues). Because the nonlinear mechanics of the cochlea is considered to be modulated by hair cell MET, nonlinear responses may offer opportunities to investigate hair cell MET.

Two-tone suppression (2TS), *i.e*., cochlear response to one tone can be reduced by the presence of another, exhibits the nonlinear nature of the healthy cochlea^29^. When a cochlea loses its sensitivity, 2TS disappears^30^. In 2TS experiments, the stimulus consists of a probe tone and a suppressor tone. Interference between the two tones causes the suppression of the probe tone response, measured at the peak responding location. 2TS has been recorded in auditory nerve responses^31–33^ and in the receptor potentials of hair cells^34,35^, as well as on cochlear mechanics^36–39^. Theoretical models have been used to investigate the underlying mechanisms for 2TS^40–42^. Recently, intracochlear pressure and potentials near the basilar membrane have been measured with two-tone stimulation^43^ and the effect of tectorial membrane attachment on 2TS has been studied theoretically^44^. These findings support the hypothesis that 2TS is caused by saturation of the OHC’s MET current and is modulated by the electromechanical feedback of the OHCs. Two decades ago, when the OHC’s MET and electromotility were less well understood, Geisler and Nuttall^38^ proposed using 2TS data to estimate the OHC’s MET transfer function. With more 2TS data and more sophisticated cochlear models, now the Geisler-Nuttall idea to obtain the intrinsic properties of MET from 2TS has become more feasible.

Some key biophysical properties of hair cell MET, such as its sensitivity, have been challenging to obtain from *ex vivo* measurements due to large variations, possibly stemming from the non-natural conditions. In 1997, Geisler and Nuttall proposed to use 2TS data of BM mechanics to characterize OHC MET^38^. The idea has not been realized because at the time of the proposal there was not enough physiological information regarding hair cell electrophysiology and OoC mechanics. However, there have been many advances since then (*e.g*., better knowledge in OHC electromotility and OoC mechanics). The Geisler-Nuttall theory and recent progress in OoC mechanics motivated us to correlate 2TS with MET operations explicitly. For this purpose, a computational cochlear model incorporating nonlinear MET channel kinetics, a fully-deformable OoC mechanics, and the fluid dynamics of the cochlea has been developed. The objective of the study is to analyze existing 2TS data with the model, more specifically, (1) to estimate the MET sensitivity using 2TS experimental data and (2) to explain the effect of the hair cell’s adaptation on 2TS temporal pattern.

## Methods

### Nonlinear model in the time domain

A nonlinear model that incorporates cochlear fluid dynamics, OoC mechanics and OHC electrophysiology was developed from a previous linear model^45^. This model takes into account the interaction between intracochlear fluid and the OoC complex, *i.e*. the OoC, the basilar membrane and the tectorial membrane. The transduction current saturates with large hair bundle deflections, which is the main nonlinear component. Unlike our previous studies that used a finite difference method in the fluid domain and a finite element (FE) method in the structural domain, a FE method was applied in both the structural and fluid domains for the new model. The global equations were solved in the time domain.

Figure 1 shows a schematic of the cochlear model. A long thin tube filled with fluid (12.1 mm long and 0.6 mm high), which represents the uncoiled gerbil cochlea, is divided into two compartments by the OoC. The upper compartment represents the scala vestibuli and scala media, and the lower one represents the scala tympani. The fluid spaces are connected through the helicotrema (Fig. 1A). As our model does not incorporate the middle ear, stimulations are applied at the oval window in the upper compartment near the basal end of the cochlea, and the round window in the lower compartment serves as a pressure release. The rest of the chamber boundaries are stationary. The *x*-, *y*- and *z*-axes correspond to the longitudinal, radial and transverse directions, respectively. The fluid domain is approximated as 2-D in the numerical simulation to save computational costs.

The structures in the OoC complex are modeled by beam elements (Fig. 1C). The radial section (Fig. 1B) is repeated every 10 µm along the cochlear length. The radial sections are coupled through longitudinal elements of the basilar membrane, the tectorial membrane, the reticular lamina and the Y-shaped structure associated with the OHC and the Deiters’ cell. The medial edge of the basilar membrane is hinged, while the lateral edge is clamped. The tectorial membrane is clamped at the spiral limbus along its medial edge, and its lateral edge is hinged to the tips of the OHC hair bundles.

The OoC complex model interacts with the active OHCs through the cells’ MET channel kinetics and electromotility (Fig. 1D). The MET current due to the OHC hair bundle deflection is the input to the electrical circuit of the OHC. The active forces generated from both hair bundle and somatic motility modulate the vibration of the OoC complex. While the fluid and structural mechanics are considered linear, nonlinearities of the OHC physiology were incorporated, such as the saturation of MET current and voltage-dependent membrane conductance^46^. We observed little effect of the latter nonlinearity on the mechanics, because under our simulated conditions the OHCs’ membrane potential did not change enough (<5 mV at CF = 17 kHz) to cause nonlinear changes in the capacitance/conductance.

### Fluid-structure interaction

After assuming the fluid is inviscid and incompressible, the Naiver-Stokes equation reduces to

1 $${\nabla }^{2}p=0,$$

where ∇^2^ is the Laplacian operator and *p* is the fluid pressure. The boundaries of the fluid domain are as follows. The pressure at the oval window was given as an input. The pressure at the round window was set equal to zero. All other boundaries were considered rigid. There are two deformable fluid-structure boundaries—the upper and lower surfaces of the OoC complex. The midpoint of the basilar membrane and the lateral end of the tectorial membrane represent the midpoint of the top and bottom fluid interacting surfaces, respectively, because those two points are approximately in the middle of the radial span. At those boundaries, the pressure gradient normal to the surface is proportional to the accelerations of the surfaces (*a*_*TM*_ and *a*_*BM*_):

2 $$\partial p/\partial z=\{\begin{array}{ll}-{\alpha }_{32}\rho {a}_{TM}, & z={z}_{TM}\\ -{\alpha }_{32}\rho {a}_{BM}, & z={z}_{BM}\end{array}.$$

Because the fluid domain is 2-D and the structural domain is 3-D, conversion factors are needed. The conversion factor *α*_32_ = 0.6 was determined by approximating the deforming pattern of the interacting surfaces along the radial direction as a half sine wave. The fluid forces *f*_*Fluid*_ on the tectorial membrane and the basilar membrane were calculated from the following equations:

3 $${f}_{Fluid}=\{\begin{array}{ll}-{\alpha }_{23}{p}_{TM}{S}_{TM}, & z={z}_{TM}\\ -{\alpha }_{23}{p}_{BM}{S}_{BM}, & z={z}_{BM}\end{array},$$

where *p*_*TM*_ and *p*_*BM*_ are the pressure differences across the fluid-structure interfaces, and *S*_*TM*_ and *S*_*BM*_ represent the *effective* interacting surface areas of the top and bottom surfaces of the OoC complex. Since radial- and longitudinal- running elements divide the TM and the BM into tiles, the effective area for a node is the sum of the areas of its 4 adjacent tiles (2 tiles for the nodes on the edges) divided by 4. The conversion factor *α*_23_ = 0.6 is to convert the fluid pressure to equivalent resultant forces acting at the lateral edge of the tectorial membrane and the centerline of the basilar membrane. The fluid within the OoC complex was not explicitly modeled. Instead, the fluid pressure inside the OoC complex was assumed to be the mean of pressure on both interfaces.

### OHC channel kinetics and electro motility

OHCs generate active forces through hair bundle (*f*_*MET*_) and somatic (*f*_*OHC*_) motility, which are dependent on the MET channel open probability (*p*_*o*_) and membrane potential (*V*_*m*_). The channel kinetics are obtained from previous studies based on the gating spring theory^47^. The MET channel’s status is governed by first-order kinetics,

4 $$d{p}_{o}/dt={k}_{CO}(1-{p}_{o})-{k}_{OC}{p}_{o},$$

where *k*_*CO*_ and *k*_*OC*_ are the rate coefficients defined from the elastic potential energy of a putative gating spring with spring constant *k*_*G*_.

5 $${k}_{CO}={A}_{0}\exp \{\gamma b{k}_{G}({x}_{HB}-{x}_{A}-{x}_{0})/(2{k}_{B}T)\},\,{\rm{and}}$$

6 $${k}_{OC}={A}_{0}\exp \{-\gamma b{k}_{G}({x}_{HB}-{x}_{A}-{x}_{0})/(2{k}_{B}T)\},$$

where *k*_*B*_ is the Boltzmann constant, *T* is the absolute temperature, *A*_0_ is a rate constant, *γ* is the geometric gain, *b* is the gating swing, *k*_*G*_ is the gating spring stiffness, *x*_*HB*_ is the hair bundle displacement, *x*_*A*_ is the bundle displacement due to adaptation, and *x*_0_ is a constant. The MET sensitivity σ near the resting open probability *p*_o,*rest*_ is defined as

7 $$\sigma =\,\gamma b{k}_{G}{p}_{o,rest}(1-{p}_{o,rest})/({k}_{B}T).$$

For instance, according to this equation, a hair cell operating near *p*_o,*rest*_ = 0.5 is 2.8 times more sensitive than operating near *p*_o,*rest*_ = 0.9.

The speed of MET adaptation is determined by a rate constant *k*_*A*_ such as

8 $$d{x}_{A}/dt={k}_{A}(\gamma {k}_{G}{x}_{HB}-{k}_{E}{x}_{A}),$$

where *k*_*E*_ is the combined stiffness of the extent springs^48^. The transduction channel introduces a force (*f*_*MET*_) proportional to the change of open probability.

9 $${f}_{MET}={f}_{MET,max}({p}_{o}-{p}_{o,rest}).$$

*f*_*MET*,*max*_ is the maximum gating force. In the FE model, *f*_*MET*_ is applied as a coupled force at the tip and root nodes of a hair bundle so that the bundle deflects in the excitatory-inhibitory direction.

The electrical potential difference between endocochlear potential *E*_*P*_ and the membrane potential *V*_*m*_ determines the MET current *I*_*MET*_, or

10 $${I}_{MET}={C}_{S}d({E}_{P}-{V}_{m})/dt+{G}_{S}({E}_{P}-{V}_{m}),$$

where *C*_*s*_ and *G*_*s*_ are the capacitance and conductance of the hair bundle, respectively. The stereocilia conductance is proportional to the maximum saturating conductance and to *p*_0_. For the basolateral membrane, it was assumed that the membrane equilibrium potential is maintained at *E*_*k*_. Then, the basolateral current *I*_*m*_ is

11 $${I}_{m}=d({C}_{m}{V}_{m})/dt+{G}_{m}({V}_{m}+{E}_{K}),$$

where *C*_*m*_ and *G*_*m*_ are the capacitance and conductance of the OHC basolateral membrane, respectively. The OHC somatic force *f*_*OHC*_ is proportional to the change of membrane potential:

12 $${f}_{OHC}={\alpha }_{OHC}({V}_{m}-{V}_{m,rest}).$$

*V*_*m*,*rest*_ is the membrane potential at rest and *α*_*OHC*_ is the electromechanical gain.

### Governing equations in matrix form

The governing equation for the fluid pressure is discretized and expressed in matrix-vector form:

13 $${{\bf{A}}}_{pp}{\bf{p}}+{{\bf{A}}}_{pa}{\bf{a}}={\bf{b}}.$$

**A**_*pp*_ corresponds to the Laplace operator. **A**_*pa*_**a** represents the boundary conditions at the OoC complex-fluid interacting surfaces, and **a** is the acceleration vector of the OoC complex interacting nodes. **b** represents the boundary conditions of other boundaries including the oval and round windows.

The OoC complex structures represented by mass (**M**), damping (**C**) and stiffness (**K**) matrices are subjected to a fluid force (**f**_*FLD*_), and two OHC active forces (**f**_*MET*_ and **f**_*OHC*_). **M** and **K** are the assembly of elemental mass and stiffness matrices, and **C** is a linear combination of **M** and **K** (Eq. 15). **f**_*FLD*_ is determined by the pressure input at the stapes and a matrix **A**_*ap*_ relating pressure to forces at structural degrees of freedom at the interacting surfaces (Eq. 16). **f**_*MET*_ and **f**_*OHC*_ are the collection of OHC active forces in each longitudinal section (Eq. 9 and 12). The global equation

14 $${\bf{M}}\ddot{{\bf{x}}}+{\bf{C}}\dot{{\bf{x}}}+{\bf{K}}{\bf{x}}={{\bf{f}}}_{FLD}+{{\bf{f}}}_{MET}+{{\bf{f}}}_{OHC},$$

is used to solve for the structural displacement vector (**x**). Rayleigh damping was used to form the damping matrix,

15 $${\bf{C}}={\alpha }_{c}{\bf{K}}+{\beta }_{c}{\bf{M}},$$

where *α*_*c*_ = 0 ms and *β*_*c*_ = 20 ms^−1^.

The fluid pressure determines the nodal force vector acting on the OoC complex surfaces:

16 $${{\bf{f}}}_{FLD}={{\bf{A}}}_{ap}{\bf{p}}.$$

Since no time derivatives of the pressure appear in the governing equations, the pressure was substituted using Eq. 13:

17 $${{\bf{f}}}_{FLD}={{\bf{A}}}_{ap}{{\bf{A}}}_{pp}^{-1}({\bf{b}}-{{\bf{A}}}_{pa}{\bf{a}}).$$

Thus, the combined governing equation for the fluid and structure domains (Eqs 13 and 14) is:

18 $${{\bf{M}}}_{EFF}\ddot{{\bf{x}}}+{\bf{C}}\dot{{\bf{x}}}+{\bf{K}}{\bf{x}}={{\bf{f}}}_{EFF}+{{\bf{f}}}_{MET}+{{\bf{f}}}_{OHC},$$

where **M**_*EFF*_ and **f**_*EFF*_ are the effective system mass and load, respectively:

19 $${{\bf{M}}}_{EFF}={\bf{M}}+{{\bf{A}}}_{ap}{{\bf{A}}}_{pp}^{-1}{{\bf{A}}}_{pa},$$

20 $${{\bf{f}}}_{EFF}={{\bf{A}}}_{ap}{{\bf{A}}}_{pp}^{-1}{\bf{b}}.$$

The governing equations were solved using Newmark’s method (see Supporting Materials)

### Computation

There are approximately 17,000 pressure nodes (1 degree of freedom per node) in the fluid domain. Since the equation that we are integrating (Eq. 18) does not include the pressure degrees of freedom, the computational cost primarily depends on the number of structural degrees of freedom (once the matrix **M**_*EFF*_ has been calculated). For the OoC complex model, there are 25 nodes per cross-section and 1201 cross-sections (30025 nodes in total, 6 degrees of freedom per node). The code was written in Matlab (Mathworks, Natick, MA), and executed on a Dell desktop (32 GB physical memory, Intel Core i7-4770 processor). Calculation of **M**_*EFF*_ and **f**_*EFF*_ took 212 and 9.0 s, respectively. With a typical time step size of 4 µs, it took 22 minutes to simulate a time-span of 1 ms. The parameters used in the numerical simulation are given in Supporting Materials (Tables S1 and S2).

## Results

**Figure 2:**
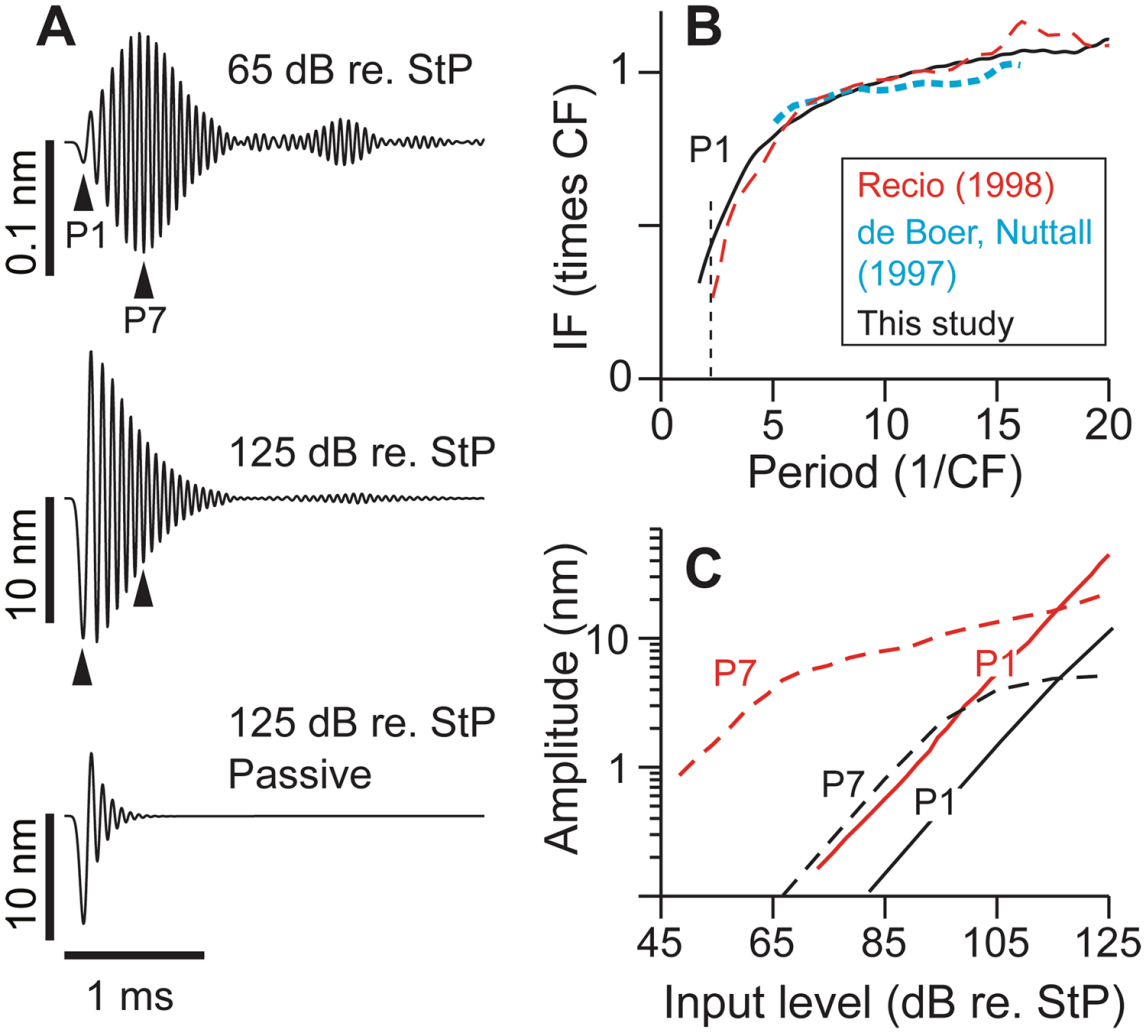
Nonlinearity in impulse responses. (**A**) Basilar membrane responses to a click. The responses are different according to stimulus level and active feedback. (**B**) Instantaneous frequency (IF) of the temporal response normalized with CF as a function of time. To compare with experimental data (dashed lines^50,51^), IF is normalized to CF and time is expressed as periods (1/CF). Experimental data are shifted horizontally to match the first peak towards scala tympani. (**C**) Basilar membrane peak amplitude as a function of input level from simulation (black). Experimental data from chinchilla (red lines^50,77^) are shown together for comparison. The amplitude of the first (P1) and seventh (P7) peak are presented. dB re. StP: input level expressed in dB with respect to 20 µPa at the stapes.

**Figure 3:**
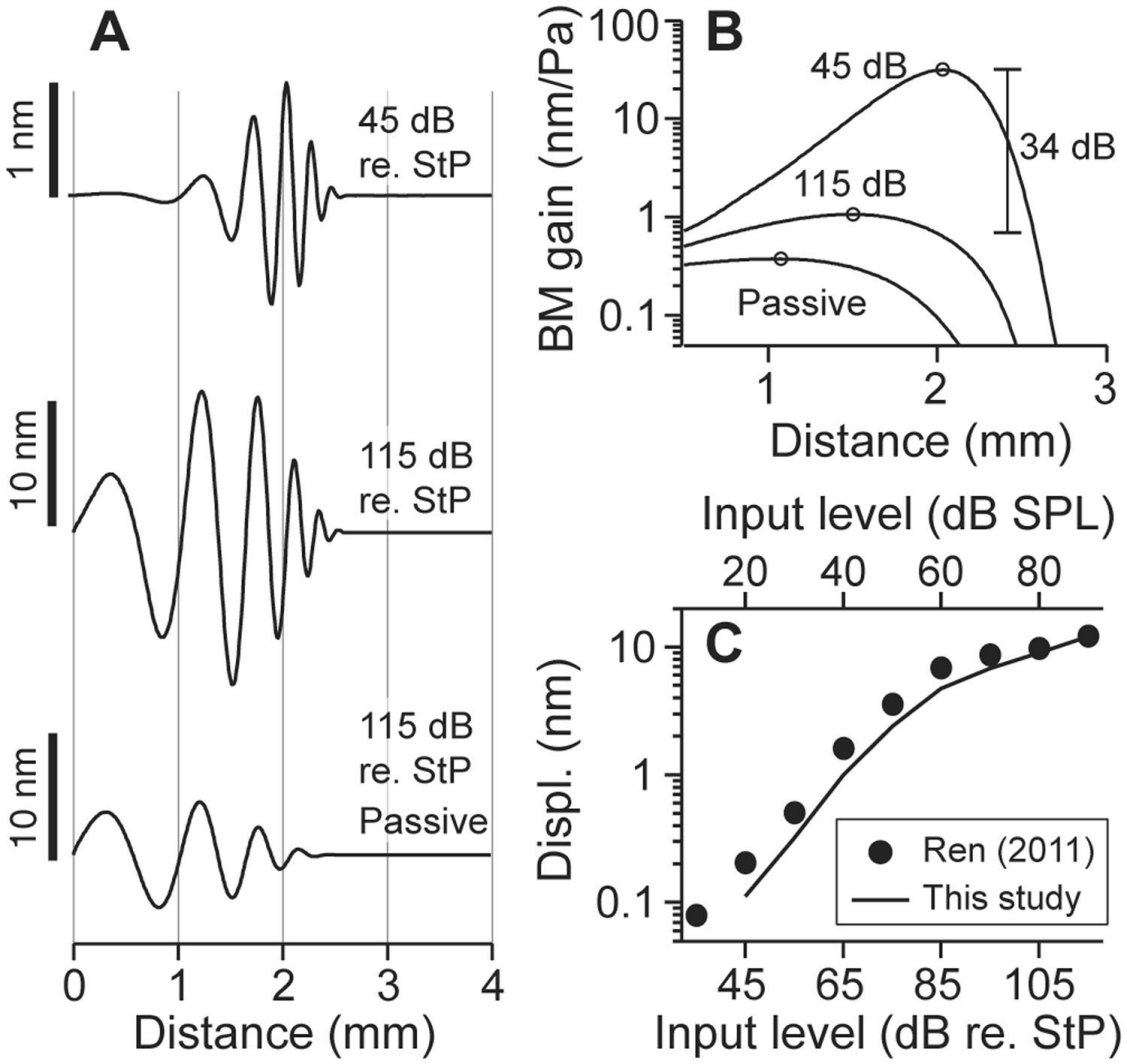
Nonlinearity in pure tone responses. (**A**) Pure tone responses at 17 kHz: spatial patterns of the traveling waves at low input level, at high input level and in passive condition (no active feedback from the OHCs). Scale bars show the displacement of the traveling waves. (**B**) Spatial envelopes from the three conditions in (**A**), normalized to the input level. (**C**) Comparison between the simulation result and experimental data from the gerbil cochlea^56^ (16 kHz tone).

**Figure 4:**
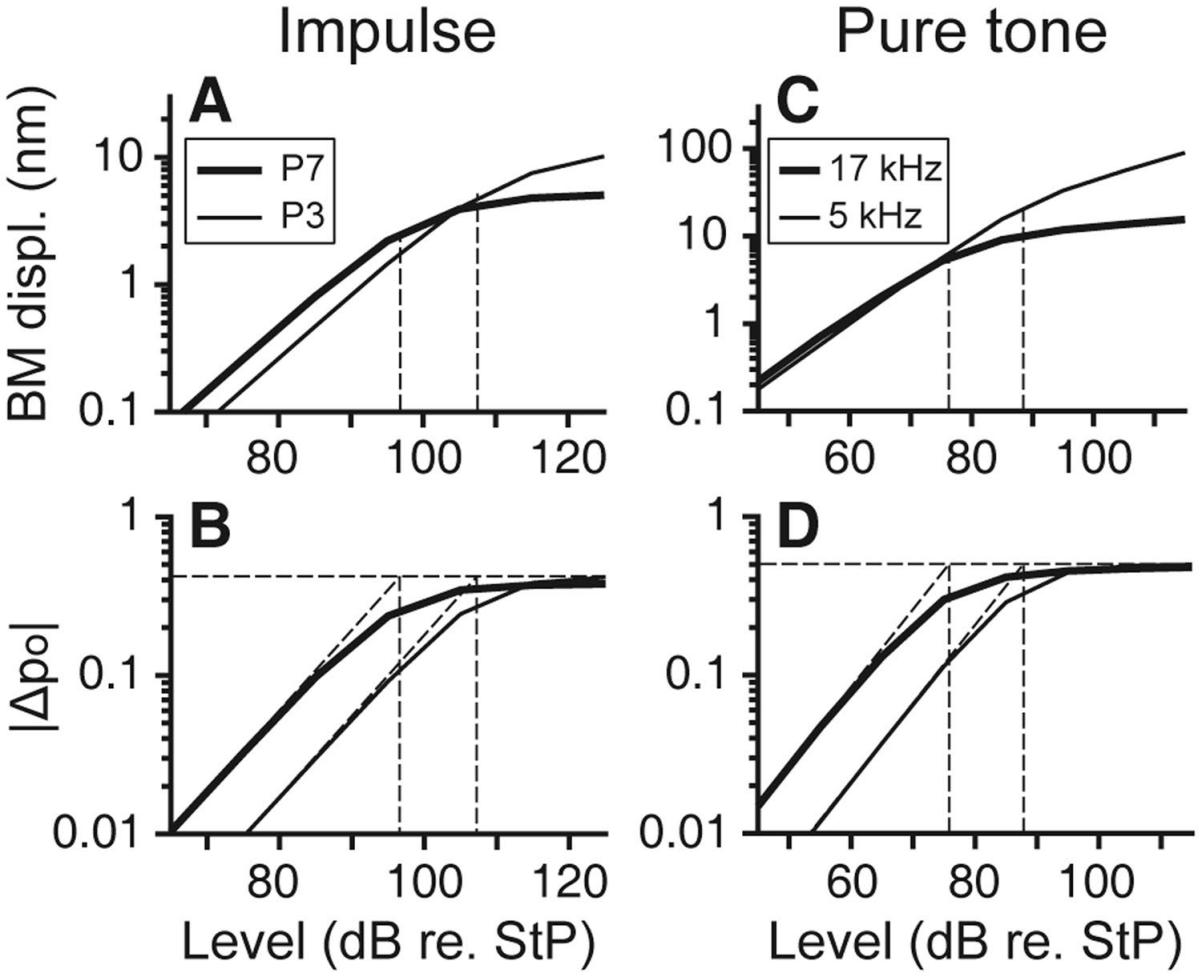
Saturation of MET results in nonlinear amplification of cochlear responses. (**A**) Impulse responses: BM displacement amplitudes of the 3rd (P3) and 7th peak (P7). (**B**) Impulse responses: MET channel open probability of the P3 and P7. Curves in panel (A) and (**B**) were obtained at *x* = 2 mm. **(C**) Pure tone responses: BM displacement amplitudes at 5 and 17 kHz. (**D**) Pure tone responses: MET channel open probability at 5 and 17 kHz. Curves in panel (C) and (**D**) were obtained at respective best responding locations: *x* = 5 mm (thin line) and *x* = 2 mm (thick line). Intersection of the linear increase and saturation shows the transition point from linear to nonlinear responses.

**Figure 5:**
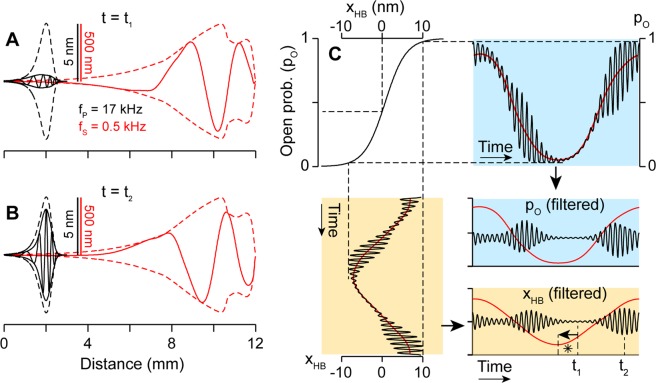
Two-tone suppression: Interaction between probe and suppressor tones. A probe tone at 17 kHz, 85 dB re. StP and a suppressor tone at 500 Hz, 120 dB re. StP were delivered simultaneously. The two components were separated and plotted in black (probe) and red (suppressor). (**A**,**B**) Spatial pattern of traveling waves along the basilar membrane when the probe tone response is most suppressed (at *t* = *t*_1_, top), and least suppressed (*t* = *t*_2_, bottom). The outlines with broken curves indicate the wave envelope when each tone is delivered individually. Note that the scales of the black and red curves are different by a factor of 100. (**C**) Input-output (x_HB_-p_O_) relationship of MET shown together with 2TS. During one cycle of suppressor tone (red), the probe tone response (black) varies in amplitude. The probe tone response is suppressed when the hair bundle is deflected toward either direction, or when the MET current saturates.

**Figure 6:**
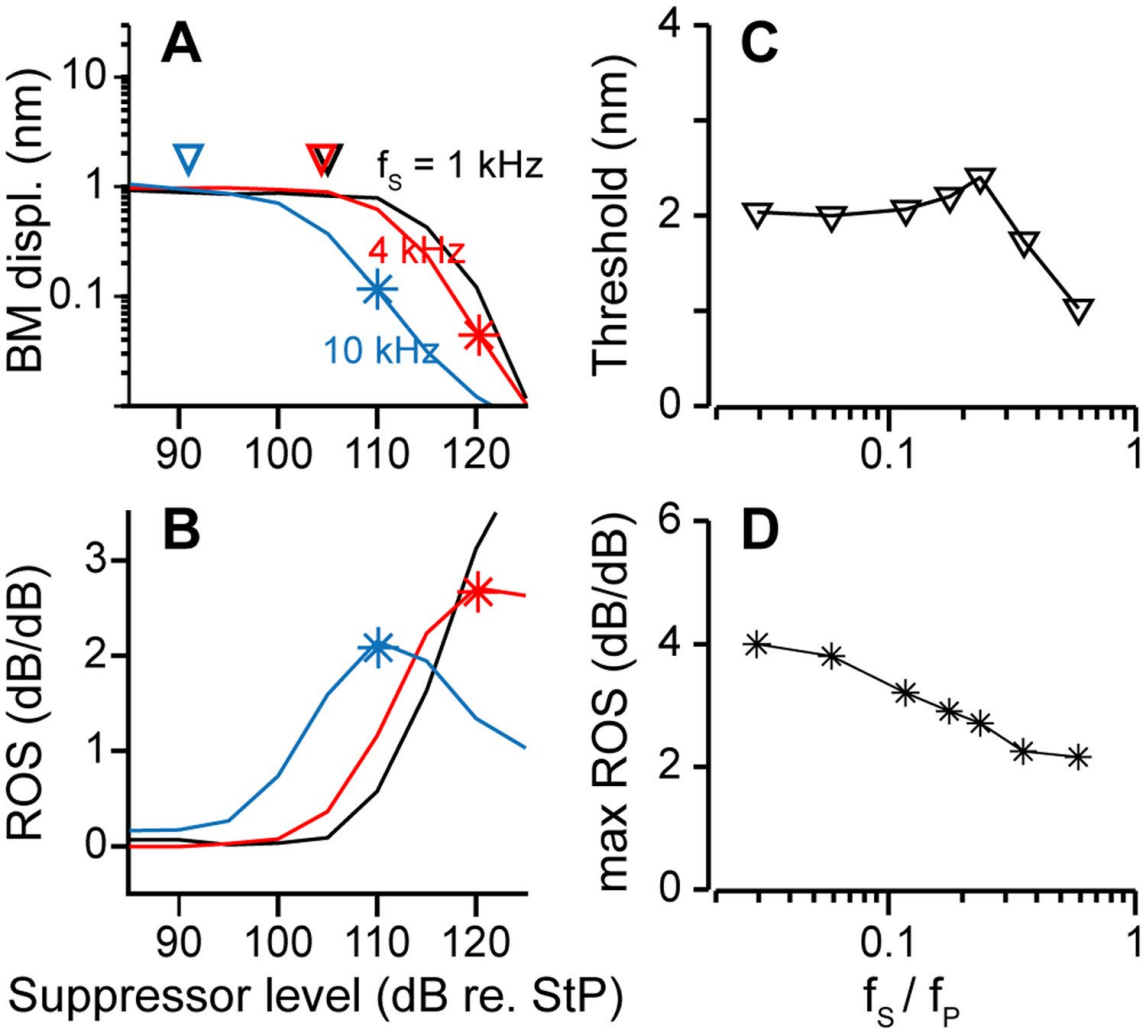
Quantifying 2TS with threshold and maximum rate of suppression While the probe tone was maintained at 17 kHz, 65 dB re. StP, different suppressor tones were applied at different SPLs, and the rate of suppression (ROS) was obtained. (**A**) BM displacement amplitude of the probe response as a function of suppressor input level. Black, red and blue lines represent responses with 1, 4 and 10 kHz suppressor tones. The threshold (▽) is defined at the BM displacement amplitude level where the probe tone amplitude decreases by 1 dB. (**B**) ROS calculated as the slope of probe tone amplitude vs. suppressor input level. Asterisks in (**A**,**B**) indicate the max ROS. (**C**,**D**) Suppression threshold amplitude and max ROS as a function of suppressor and probe frequency ratio (*f*_*S*_/*f*_*P*_). All simulation results were taken at the 17 kHz best frequency location.

**Figure 7:**
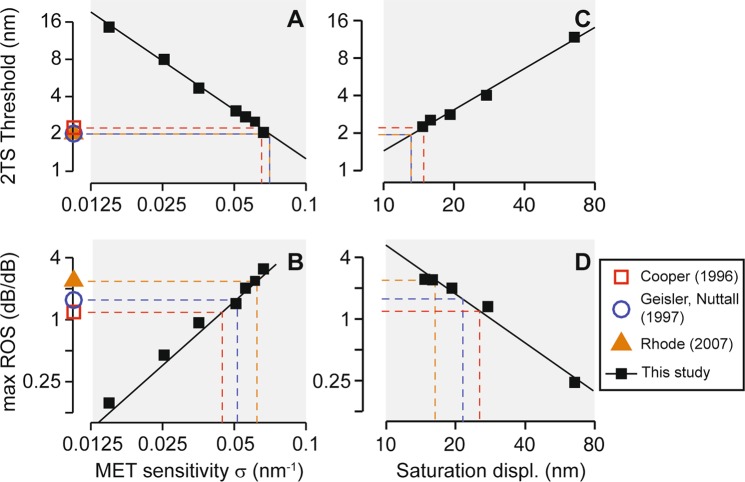
Estimation of the MET sensitivity with 2TS measurements. (**A**,**B**) 2TS threshold amplitude and ROS predicted as a function of the MET sensitivity. (**C**,**D**) 2TS threshold amplitude and ROS predicted as a function of 90% saturation displacement of the hair bundle with constant current sensitivity. Solid lines are the functions fitted to simulation results (■). Colored symbols represent weighted average experimental data from three studies^38–40^. Dashed lines show the estimated MET sensitivity and saturation displacement from existing measurements of threshold amplitude and max ROS. Simulation results were taken at the 17 kHz best frequency location.

**Figure 8:**
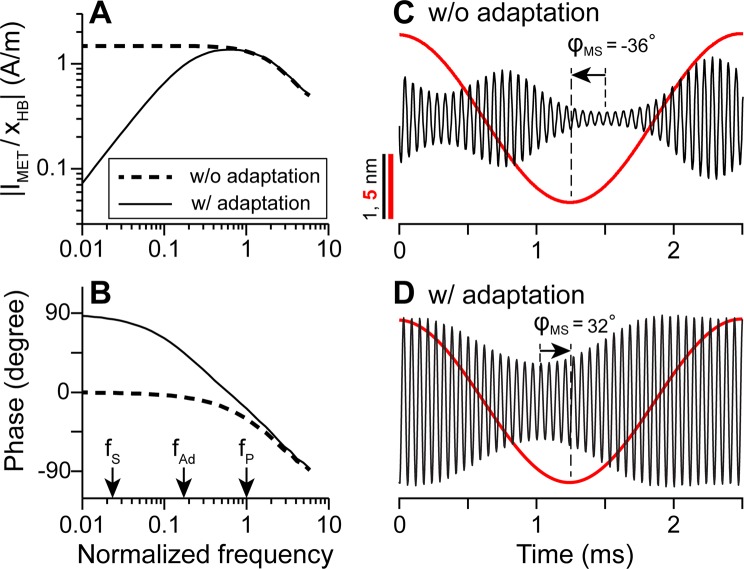
Effect of adaptation on temporal patterns of 2TS. (**A**,**B**) MET transfer function and phase relation at 17 kHz best-frequency location. Frequency is normalized to CF. Arrows indicate the probe (*f*_*P*_) suppressor (*f*_*S*_), and adaptation cut-off frequency (*f*_*Ad*_). Solid and dashed lines show simulation results with and without the adaptation, respectively. (**C**,**D**) Simulated temporal pattern of probe tone response (black, 65 dB re. StP) within one cycle of suppressor tone (red, 115 dB re. StP) without and with the adaptation, respectively. The arrows indicate the phase of maximum suppression of probe tone with respect to the suppressor tone (${\varphi }_{{\rm{MS}}}$).

**Figure 9:**
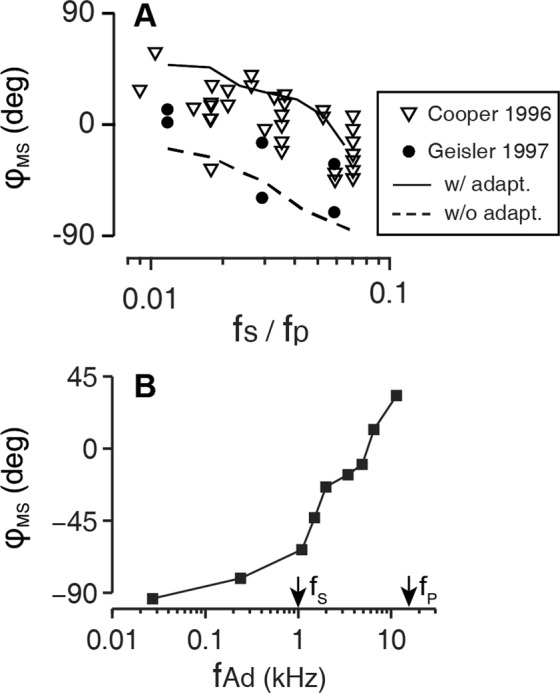
Phase of maximum suppression (${\varphi }_{{\rm{MS}}}$) and MET adaptation. (**A**) The suppressor frequency is normalized to the probe frequency. The simulation results are shown for two cases: with (solid line) and without adaptation (dashed line). Experimental data were from two studies^37,38^. (**B**) ${\varphi }_{{\rm{MS}}}$ as a function of adaptation cut-off frequency with probe tone at 17 kHz and suppressor tone at 1 kHz.

To validate our model, we compared both pure tone and impulse responses with existing measurements. It should be noted that our model presents the gerbil cochlea, while referenced experimental data are a cohort of different rodent species (gerbil, chinchilla, and guinea pig). To summarize, nonlinear characteristics observed in impulse (Fig. 2) and pure-tone (Fig. 3) responses were simulated. In Fig. 4, we demonstrate how the saturation of OHC MET can explain the cochlear nonlinearity. Temporal and spatial patterns of 2TS were analyzed to obtain two measured quantifies of 2TS: suppression thresholds and rates (Figs 5 and 6). We show that these two 2TS quantities and the MET sensitivity are monotonically related (Fig. 6). This monotonic relationship made it possible to exploit experimental data to estimate the MET sensitivity (Fig. 7). The timing of the maximum suppression was explained with MET adaptation (Figs 8 and 9).

### Nonlinear characteristics of cochlear mechanics in response to impulse and pure tone

Impulse responses at a basal location (approximately 2 mm from the basal end of the gerbil cochlea) were simulated and compared to experimental data (Fig. 2). Because middle ear transmission was not included in the model, simulation input levels were expressed in dB with respect to 20 µPa at the stapes, hereafter written as “dB re. StP”. For a rough comparison with experimental data, a 25 dB middle ear gain^49^ was assumed. That is, 0 dB SPL roughly corresponds to 25 dB re. StP. The basilar membrane responses to a 20 µs click and a 17 kHz tone at 65 dB re. StP show characteristics observed in experiments (Figs 2 and 3). For an impulse response, the main response lobe lasts for approximately 1 ms and the response amplitude initially increases and then gradually decreases. The basilar membrane oscillated for more than a dozen cycles before decaying below 5% of the peak displacement. Such a large number of oscillatory cycles, comparable to experimental measurements^50,51^, is a signature of highly tuned systems. Secondary lobes were considered another sign of a sensitive cochlea since they were absent in cochleae that were surgically damaged or overstimulated^50,52^. The amplitudes of the secondary lobes became smaller as the input level increased as was shown in other modeling studies^53,54^. When passive, the first peak was the greatest and the oscillations died away (below 5% of the first peak) within six cycles.

A change of instantaneous frequency (IF) with time, termed the frequency glide^51^, has been observed in cochlear impulse responses^55^. At basal cochlear locations, the IF at the onset of the response is lower than the characteristic frequency (CF) and gradually increases with time (Fig. 2B). When normalized with the CF, the simulation result compares well with the experimental data from the 18 kHz CF location of the guinea pig cochlea^51^ and the10 kHz best frequency location of the chinchilla cochlea^50^.

Level-dependence is another well-known characteristic of cochlear physics. For example, responses to small sounds are amplified and tuned more than the responses to loud sounds. Our model reproduces observed characteristics. In a time-domain response (Fig. 2A), this level-dependent amplification/tuning appears as increased number of oscillations when the SPL is small. When the cochlea is passive, the basilar membrane oscillates only a few cycles after the impulse and the first peak is the greatest. On the contrary, when the active cochlea is subjected to small sounds, it vibrates a few dozen cycles. The amplitude of the initial peak in an impulse response increases linearly regardless of the input level (Fig. 2C). For later peaks, the slope of the response-SPL curve decreases at high input levels (>95 dB re. StP). The simulated response of the 7^th^ peak shows a similar level of nonlinearity (in the slope of the amplitude vs. input level) as compared to experimental data (Fig. 2C). The lowest growth rate is 0.11 and 0.15 dB/dB from the simulation and experimental data^50^, respectively. The peak amplitude of the simulated response was 10 to 20 dB smaller than the experimental data. Such differences are ascribed to different species and best frequency locations (17 kHz in gerbil versus 10 kHz in chinchilla).

The level-dependence is also observed in pure tone responses. In Fig. 3, the model responses to a pure tone (17 kHz) at different stimulation levels are shown. Traveling wave patterns at low SPL (45 dB re. StP), high SPL (115 dB re. StP) and in a passive condition (no OHC active forces) are shown in Fig. 3A. As the input level increases, the hair cell’s MET saturates over a greater span (see Supporting Materials Fig. S2). Thus, the amplification due to OHC active feedback decreases and the spatial envelope asymptotes to the passive condition (Fig. 3B). The peak location shifts towards the base as the stimulation level increases, consistent with experimental observations (circles on the curves in Fig. 3B). Simulated basilar membrane displacement at the best frequency location is comparable to a measured result (Fig. 3C). At 115 dB re. StP, the experimental data still shows compressive nonlinearity, which indicates OHC feedback is not yet saturated. The response amplitude gain (Fig. 3C) is comparable to measurements at the basal turn of the gerbil cochlea^56^. Although not presented in the plot, the model response became linear when the stimulation level was >145 dB re. StP. Responses at a more apical location are shown in the Supporting Materials (Fig. S1).

These nonlinear responses originate from nonlinear MET. Displacement amplitudes of impulse and pure tone responses and the amplitude of MET response as a function of the input level were plotted in Fig. 4. The MET response was represented by the amplitude of MET channel open probability (|∆*p*_O_| in Fig. 4). For both pure tone and impulse responses, the displacement amplitudes are linear at low SPLs, but becomes compressed at higher levels due to saturation of the MET current (Fig. 4A,C). That is, the slope is 1 dB/dB when the stimulus level is <65 dB re. StP, and is <1 dB/dB for higher stimulus levels. The intersection of the two tangential lines taken at the lowest and highest stimulus levels defines the critical level, which indicates the transition from linear to nonlinear growth in the response amplitude (Fig. 4B,D). For an impulse response, because it takes time for the OHC feedback to develop, the displacement amplitudes of later peaks show stronger nonlinear effects than the ones right after the onset. The nonlinear response indicates the overall effect of MET saturation of the OHCs around the peak responding location. While the MET current of some OHCs is saturated at the peak location, there is room for those OHCs off the peak region to increase the MET current. Also, the energy coming from fluid-structure interaction becomes dominate as the MET is saturated. Therefore, the nonlinearity in amplitude curves are less dramatic than the nonlinearity of a single OHC’s MET.

### Two-tone suppression with low-side suppressors

An example case of 2TS was simulated, and its temporal and spatial patterns are presented in Fig. 5. In 2TS, if the suppressor frequency is lower than that of the probe tone, it is called a low-side suppressor. The probe tone response exhibits both tonic (overall averaged suppression) and phasic (in a certain phase relationship with the suppressor tone) suppression with low-side suppressors^38^. The maximum suppression of the probe tone occurs near the largest displacement of the suppressor tone towards the scala tympani (Fig. 5C). The asymmetry of maximum suppression suggests that the hair cell MET saturates more readily when the hair bundle is deflected toward its inhibitory direction. The simulated asymmetry occurs when the resting *p*_*o*_ is less than 0.5 (*e.g*., the model used *p*_*o,rest*_ = 0.4). When *p*_*o*_ > 0.5, the maximum suppression occurs while the suppressor tone deflects the basilar membrane toward the scala vestibuli. There is a secondary suppression while the basilar membrane is displaced towards the scala vestibuli side. Snapshots of the basilar membrane traveling waves (Fig. 5A,B) were taken when the hair bundle deflection was most (*t* = *t*_1_) and least (*t* = *t*_2_) suppressed. The amplitude of the probe tone response is reduced by 90% and 20% compared to the unsuppressed state in the two cases. The shape of probe tone traveling waves changes depending on suppressor tone level: The shape (envelope) of probe tone traveling waves at the most suppressed state is similar to the shape of pure tone traveling waves at high SPL, while the least suppressed situation is close to pure tone responses at low SPL (Figs 3A and 5B). This change of response shape due to suppressor tone level occurs because the least and most suppressed timings correspond to the least and most sensitive states of MET operation, respectively.

Following the Geisler-Nuttall idea^38^ (their Fig. 11), the MET transfer function is shown together with 2TS in Fig. 5C. The decreased MET sensitivity due to large suppressor displacement reduced the active feedback of OHCs, thus the MET current of the probe tone became ‘phasic’—the probe tone response amplitude changes depending on the phase of the suppressor tone. Note that the maximum suppression does not coincide with the peak of the suppressor tone (*In Fig. 5C).

### The proximity between two tones affects the extent of suppression

According to the literature, the extent of suppression depends on the proximity between the two tones (represented by the frequency ratio), and the level of the suppressor tone^36,37^. To quantify the extent of suppression, 2TS with suppressors at various frequencies and levels were simulated. The probe tone response is represented by the amplitude of the basilar membrane, corresponding to the probe tone frequency component. The probe tone response decreases as the suppressor level increases (Fig. 6A). For the suppressor tone to affect the probe tone response, the suppressor tone must be loud enough to affect the MET sensitivity in the region of the probe tone response. There exists a threshold level below which the suppressor tone does not affect the probe tone response. Following Rhode and Cooper^36^, the threshold is defined as the suppressor level (represented by the basilar membrane displacement amplitude) at which the probe response is suppressed by 1 dB. The frequency dependence of the threshold is shown in Fig. 6C. The threshold is 2 nm when the frequency ratio (*f*_S_/*f*_P_) is <0.15. As the suppressor frequency (*f*_S_) increases, the threshold remains at a certain level and then quickly rolls off. This characteristic is consistent with experimental observations which show that the threshold amplitude is nearly constant when the suppressor frequency is one octave below the CF and that the threshold decreases as the suppressor frequency approaches the CF^39,57^.

The rate of suppression (ROS), the slope of the curve in Fig. 6A, is another quantity used to characterize 2TS (Fig. 6B). At low SPLs (<65 dB re. StP), the probe amplitude remains unchanged and the ROS is zero. The suppression becomes most effective when the suppressor increases to a certain level. ROS peaks at a certain suppressor level (Fig. 6B). The maximum rate of suppression (max ROS) decreases as the frequency ratio (*f*_S_/*f*_P_) increases (Fig. 6D). Similar ROS patterns were reported in auditory nerve responses^33^. Here the suppressor level is not limited (e.g., max ROS occurs at >125 dB re. StP with 1 kHz suppressor). In experiments, however, the highest suppressor level hardly exceeded 100 dB SPL. To compare with experimental data, max ROS was calculated with an upper limit of 120 dB re. StP on the suppressor level in the following sections.

### Estimating the MET sensitivity from 2TS threshold and maximum rate of suppression

2TS data provide a means to investigate hair cell MET because the 2TS occurs as a result of saturating (nonlinear) MET. We simulated the effect of the MET sensitivity on the probe tone response (17 kHz, 65 dB re StP). To adjust the MET sensitivity, the value of *b* in Eqs 5 and 6 was increased or decreased.

The threshold and max ROS were obtained for different levels of the MET sensitivity (Fig. 7A,B). The MET sensitivity, defined by Eq. 7, has the physical unit of nm^−1^. In the present model, the MET sensitivity varies from 0.08 nm^−1^ (basal end) to 0.02 nm^−1^ (apical end), and the change of sensitivity applies to all locations (multiplied by the same factor). As the MET sensitivity increased, the suppressor displacement required to saturate the MET current became smaller. That is, as MET became more sensitive, the 2TS threshold decreased monotonically. At two asymptotic cases, where the MET sensitivity is zero and infinite, the threshold amplitude should approach infinite and zero, respectively. Also, the slope of the current-displacement curve changed more dramatically over a narrower displacement range with a higher MET sensitivity, resulting in an increase of the ROS. The simulation results were curve-fitted in terms of the MET sensitivity (σ) at the best frequency location of the probe tone, or *x* = 2 mm, using simple powerlaw functions: Threshold = 0.062 σ^−1.31^ and max ROS = 718 σ^2.06^. When σ was greater than 0.075 nm^−1^, the model became unstable. For a given 2TS threshold and max ROS, the inverse functions were used to estimate the MET sensitivity.

2TS threshold from Cooper *et al*.’s measurements^37^ ranged between 1 and 5 nm with a logarithmic mean of 2.2 nm (n = 9). Geisler and Rhode^38,39^ both reported a threshold of 2 nm. When compared to our simulations, these threshold values corresponded to a MET sensitivity of 0.065–0.070 nm^−1^ (Fig. 7A). Max ROS were derived from the probe amplitude-suppressor level curve if not provided directly. Cooper^37^ reported max ROS at around 1 dB/dB. Geisler and Nuttall^38^ presented similar results. More recent measurements showed max ROS at 2 dB/dB^57^ and at least 2.5 dB/dB^39^ at the 8 and 6 kHz CF locations. When compared to our simulations, these max ROS values were equivalent to a MET sensitivity between 0.045 and 0.060 nm^−1^ (Fig. 7B). The MET sensitivity obtained from the threshold amplitude was slightly higher than the upper limit obtained from the ROS (only 1–2 nm threshold data included).

Since we defined the MET sensitivity using channel open probability, varying σ would change both the current sensitivity (defined as the slope of MET current vs. hair bundle displacement curve) and the saturation displacement (the hair bundle displacement required for 90% saturation). Here we show that 2TS characteristics are mainly determined by the saturation displacement. To isolate the effect of saturation, 2TS responses were simulated with constant current sensitivity (Fig. 7C,D). This means that a lower MET sensitivity was compensated by a larger MET current. Saturation displacement was estimated to be 13–15 nm with threshold and 16–24 nm with max ROS (comparable to 15–22 nm derived from the MET sensitivity estimated in Fig. 7A,B).

### Temporal pattern of 2TS explained by MET adaptation

The temporal pattern of low-side 2TS is modulated by the time course in active processes. The activation of the MET channel results in a phase-lag of the MET current (low-pass filter), while adaptation results in phase-lead (high-pass filter)^58^. Together the channel activation and adaptation form a band-pass filter, whose bandwidth is determined by the cut-off frequencies of the activation and the adaptation (Fig. 8A,B). As a consequence of the adaptation, the MET current (*I*_*MET*_) can lead the hair bundle (*x*_*HB*_) deflection by 90 degrees at low frequencies (Fig. 8B). When there is no adaptation, *I*_*MET*_ and *x*_*HB*_ are in phase at low frequencies.

If the suppressor frequency is lower than the cut-off frequency of the adaptation, the effect of the high-pass filtering will be prominent in the temporal pattern, especially for the phasic suppression (Fig. 8C,D). In the present model, the adaptation cut-off frequency *f*_*Ad*_ is 4 kHz at the CF location of the probe tone. For a suppressor at 400 Hz, the adaptation lowered MET current gain to about 10% of its maximal value, reducing suppression to the extent that secondary suppression was absent. Because the MET current leads the bundle displacement at low frequencies due to adaptation, the maximum suppression can occur prior to the maximum suppressor displacement. That is, due to adaptation, the phase of maximum suppression (*ϕ*_*MS*_) can be greater than 0 (Fig. 8D).

According to measurements, maximum suppression occurred either before or after the maximum suppressor displacement^37,38^. The phase of maximum suppression is indicated as *ϕ*_MS_ in Fig. 8. The phase is dependent on the relationship between the probe (*f*_*P*_), suppressor (*f*_*S*_), and adaptation cut-off frequency (*f*_*Ad*_).

The effect of the frequency ratio on the suppression phase was analyzed when the adaptation cut-off frequency remained at 4 kHz (Fig. 9). Positive *ϕ*_*MS*_ was obtained when the frequency ratio <0.05. In contrast, *ϕ*_*MS*_ was negative when there was no adaptation, independent of *f*_*S*_/*f*_*P*_ (Fig. 9A, solid and broken curves). *ϕ*_*MS*_ increases as the frequency ratio decreases in both cases, consistent with experimental observations^37,38^. When the two frequencies are close enough (*f*_*S*_/*f*_*P*_ > 0.1), tonic suppression dominates the response and phasic modulation is shadowed: with fewer cycles of probe tone vibration within one cycle of the suppressor tone, it is difficult to determine the timing of maximum suppression accurately. Temporal responses of 2TS with 1.3, 1.5, and 1.7 kHz suppressors are demonstrated in Supporting Materials (Fig. S3).

In the other set of simulations (Fig. 9B), the effect of adaptation frequency (*f*_Ad_) on the suppression phase (*ϕ*_MS_) was analyzed. To our knowledge, there is no *in vivo* measurement of the adaptation time constant at the base of the cochlea. While probe and suppressor frequencies were fixed, *f*_Ad_ was changed from 30 Hz to 12 kHz. *ϕ*_MS_ increased as *f*_Ad_ increased. The effect of the high-pass filtering by MET adaptation is twofold: (1) MET current leads the hair bundle deflection by 90 degrees, which adds 90 degrees to *ϕ*_MS_ (see the shift between the solid and broken lines in Fig. 9A); (2) the MET gain for the suppressor decreases, resulting in less suppression (Fig. 8C,D). Geisler and Nuttall^38^ showed that *ϕ*_MS_ is also dependent on the level of suppression (stronger suppression causes more delay). Our results indicate that positive *ϕ*_MS_ is more likely to be observed when *f*_*Ad*_ ≫ *f*_*S*_ (Fig. 9B). Measured *ϕ*_MS_ is best explained when *f*_*Ad*_ is between 1 and 10 kHz. This adaptation speed is consistent with whole-cell patch clamp measurement results of the rodent outer hair cells^25,59,60^. To conclude, the phase lead of suppression with respect to suppressor displacement can be explained when the MET adaptation affects the 2TS.

## Discussion

### Nonlinearity in cochlear model studies

There have been theoretical studies regarding cochlear nonlinearity. The source of nonlinearity is becoming more specific as more is known about the active feedback from the OHCs. In the 1970s, before the active feedback from the OHCs was discovered, pioneering nonlinear models^9,61,62^ used nonlinear damping imposed on the basilar membrane to explain the mechanical nonlinearity first observed by Rhode^4^. Geisler^63^ incorporated amplitude factors to limit displacement-proportional OHC motile forces, so that the model could account for 2TS. Steele, Puria and their colleagues^42,64^ modeled the nonlinear force of the OHCs in the form of a feed-forward gain factor to reproduce 2TS and the distortion product. Verhulst and Shera^65^ used a level-dependent basilar membrane impedance that saturates at high SPL. Liu and Neely^66^ considered the sensitivity of MET as a nonlinear function of the displacement and velocity of the reticular lamina. Meaud and Grosh^67^ used the first-order Boltzmann function to represent the OHC mechano-transduction. This nonlinear mechano-transduction was combined with an OHC somatic electromotility model, analogous to a piezoelectric circuit.

Our model is similar to Meaud-Grosh’s in that the OHC MET is explicitly described. Compared to other studies, our cochlear model incorporates more detail regarding the OoC micromechanics (Supporting Materials, Tables S1 and S2). The model reproduced signature nonlinear responses in cochlear mechanics, such as a broadened response envelope with increasing stimulus level and the frequency glide (Figs 2 and 3). Taking advantage of the details in MET and micro-mechanics, in this study, we used our model to explore the MET properties of the OHC by analyzing existing data of 2TS.

### Limitations of this study

There are some limitations of our study. First, assorted data from different locations, frequencies and species were used for comparison. For example, in the literature, different probe/suppressor frequencies and levels were used to measure 2TS responses from different rodent species. While our study is focused on a specific location (*x* = 2 mm) of the gerbil cochlea, the probe tones in experiments ranged from 6 to 28 kHz. The MET sensitivity and level of nonlinearity may vary depending on the location. Second, the model details present an opportunity and a challenge at the same time. Due to the large parameter space, there can be different combinations of model parameters that could generate similar results. Although most model parameters are constrained by existing physiological data (such as geometry, mechanical properties of the basilar and tectorial membranes, etc.), in many cases, some literature data have large variations.

### The MET sensitivity estimated from 2TS

Existing 2TS data are consistent with a MET sensitivity of 0.06 nm^−1^ at the base of the cochlea (CF near 17 kHz, Fig. 7). According to electrophysiological measurements of isolated hair cells^26,59,68–70^, the MET sensitivity ranges from 0.0033 to 0.045 nm^−1^ for hair cells in rodent cochleae. Most of the single cell measurements gave roughly one order of magnitude smaller MET sensitivity than our estimation. The discrepancy can be ascribed to two aspects.

First, *in vitro* experimental limitations. Experimental preparation may alter physiological conditions of hair bundle excitation due to removal of the tectorial membrane from the OoC complex. Fettiplace and Kim^20^ demonstrated that with different stimulating methods, the current-displacement curve slope can be changed by an order of magnitude. Nam *et al*.^21^ showed both glass probe and fluid jet stimulation may cause splay between stereociliary columns and result in underestimation of the sensitivity. Often in experiments, the calcium concentration (>1 mM) is higher than that in the endolymph^71^. Beurg *et al*.^59^ showed that higher calcium concentration broadens the operating range of a MET channel. In experiments, the speed of the stimulation system is limited^25^, which may have a time constant larger than that of the channel activation. Thus, the recorded MET current may be reduced by adaptation with either step or low-frequency sinusoidal stimulation.

Second, the MET sensitivity measurement data and 2TS data used for the estimations were not all from the same species or location. Most of the sensitivity measurements were from mouse or rat cochleae and hair cells used for recordings were located at the apical turn (CF ~4 kHz), while our estimation is at the high-frequency (17 kHz) basal location and 2TS data are from guinea-pig and chinchilla cochleae (CFs ranging from 6–26 kHz). The estimation depends on available data from 2TS, thus more 2TS measurements will help us to probe MET properties accurately. Variation of the MET sensitivity at different locations and among different species could be a future study.

### Time courses affecting maximum suppression phase

For the low-frequency suppressor, maximum suppression of the probe tone would lag the maximum displacement of the suppressor given the time courses needed for the OHC active feedback to affect basilar membrane vibration. Channel activation, the RC constant of the OHC membrane, and viscoelastic delay were the sources for the delay in the simulation. The relative contribution of each component depends on the rate constant *A*_0_ in Eqs 5 and 6, conductance and capacitance of the OHC in Eq. 11, and mechanical properties of the basilar membrane (Supporting Materials, Tables S1 and S2). Some of the parameters may not be completely accurate, yet the fact that these processes result in phase delay remains.

Although adaptation has been commonly observed in excised cochlea or hair cells^25,26,58,72,73^, there is no direct evidence of adaptation in mammalian hair cells under physiological conditions. However, in experimental data that showed positive phase, such delay is most likely compensated through adaptation of MET for the suppressor tone, since MET adaptation may cause up to a 90-degree phase lead at low frequencies (Fig. 8B). Geisler^38^ also reported level-dependent changes of the *ϕ*_MS_. Less suppression due to smaller transduction currents of the suppressor component also helps to shift the phase towards the positive direction.

### MET adaptation estimated from 2TS temporal pattern

We used two extreme cases for simulation, one without adaptation and one with complete adaptation (MET current decays to zero given enough time). In practice, both extent and time constant of adaptation affect the phase shift (Supporting Materials Fig. S5). With lower adaptation extents and larger time constants, the phase falls within the two bounds in Fig. 9A. The linear fit (both phase and frequency are on a linear scale) to the data from Cooper’s study^37^ gives a zero-frequency intercept of ~20 degrees, less than 90 degrees with complete adaptation. This suggests partial adaptation at the base in a natural condition.

Some studies^74,75^ suggested adaptation in hair cells helps to maintain mechanotransduction sensitivity and to widen its dynamic range. Ricci *et al*.^58^ claimed that it works as a “prefilter” to increase the signal to noise ratio. Others^25,28,76^ argued that adaptation may contribute to frequency tuning and amplification. We did a parametric study to see how different adaptation extents and time constants affect tuning and amplification at the basal location. No significant change was seen to support its role for amplification/tuning enhancement. However, we cannot exclude the possibility that adaptation may work as a secondary filter at the apical region, where the CF is lower and mechanical tuning is broader.

### Bias of rest open probability and polarity of suppressor displacement at maximum suppression

It is still controversial whether maximum suppression occurs most closely with maximum displacement of the suppressor towards the scala tympani or the scala vestibuli. Some studies showed maximum suppression on the scala tympani side^36–38^, while others reported maximum suppression scala vestibuli side^30^ and the rest exhibited both^39^. The asymmetry between maximum and secondary suppression has been attributed to the bias of the resting open probability^38,44^. We confirmed this by showing examples of temporal patterns with open probability set at 0.4 and 0.6. When the open probability is less than 0.5, suppressor displacement at the scala tympani side results in greater suppression. Above 0.5, this trend flips (Supporting Materials Fig. S4). By studying the relative magnitude between maximum and secondary suppression, we can possibly predict the resting open probability. Unfortunately, there is no agreement in experimental data yet. If the resting open probability is close to 0.5, even small perturbations in experiments, resulting in a DC offset of the OoC complex, can alter the temporal patterns.

### Conclusion

A computer model of the cochlear mechano-transduction was created incorporating detailed OoC mechanics and nonlinear kinetics of hair cell MET. Using the model, we showed that 2TS data can be interpreted and analyzed to investigate the hair cell MET. The estimation of a key parameter – the MET sensitivity at the basal cochlea (0.06 nm^−1^) through comparison between simulation results and experimental data of 2TS helps to gain new insights about the sensory cell. The temporal pattern of 2TS represented by *ϕ*_MS_ could be explained with the hair cell’s MET adaptation.

## Supplementary information

Supporting Materials

## Data Availability

Simulation data provided in this study are available from the corresponding author on reasonable request.

## References

[1] Ashmore JF, Geleoc GS, Harbott L (2000). Molecular mechanisms of sound amplification in the mammalian cochlea. Proc. Natl. Acad. Sci. USA.

[2] Olson ES, Duifhuis H, Steele CR (2012). Von Békésy and cochlear mechanics. Hearing research.

[3] Ren T (2002). Longitudinal pattern of basilar membrane vibration in the sensitive cochlea. Proceedings of the National Academy of Sciences.

[4] Rhode WS (1971). Observations of the Vibration of the Basilar Membrane in Squirrel Monkeys using the Mössbauer Technique. The Journal of the Acoustical Society of America.

[5] Cooper NP, Rhode WS (1992). Basilar membrane mechanics in the hook region of cat and guinea-pig cochleae: Sharp tuning and nonlinearity in the absence of baseline position shifts. Hearing Research.

[6] Robles L, Ruggero MA, Rich NC (1986). Basilar membrane mechanics at the base of the chinchilla cochlea. I. Input–output functions, tuning curves, and response phases. The Journal of the Acoustical Society of America.

[7] Von Békésy, G. *Experiments in Hearing*. (McGraw-Hill, 1960).

[8] Allen JB (1980). Cochlear micromechanics–a physical model of transduction. J Acoust Soc Am.

[9] Hubbard AE, Geisler CD (1972). A hybrid-computer model of the cochlear partition. J Acoust Soc Am.

[10] Dallos P (2003). Organ of Corti kinematics. J Assoc Res Otolaryngol.

[11] Chen F (2011). A differentially amplified motion in the ear for near-threshold sound detection. Nat Neurosci.

[12] Lee HY (2015). Noninvasive *in vivo* imaging reveals differences between tectorial membrane and basilar membrane traveling waves in the mouse cochlea. Proc Natl Acad Sci USA.

[13] He, W., Kemp, D. & Ren, T. Timing of the reticular lamina and basilar membrane vibration in living gerbil cochleae. *Elife* **7**, 10.7554/eLife.37625 (2018). 10.7554/eLife.37625 PMC6125122 30183615

[14] Furness DN, Hackney CM (1985). Cross-links between stereocilia in the guinea pig cochlea. Hear Res.

[15] Beurg M, Fettiplace R, Nam JH, Ricci AJ (2009). Localization of inner hair cell mechanotransducer channels using high-speed calcium imaging. Nat Neurosci.

[16] Liberman MC (2002). Prestin is required for electromotility of the outer hair cell and for the cochlear amplifier. Nature.

[17] Ashmore J (2008). Cochlear Outer Hair Cell Motility. Physiological Reviews.

[18] Fettiplace, R. In *Comprehensive Physiology*. (John Wiley & Sons, Inc., 2017).

[19] Zheng, J., Madison, L. D., Oliver, D., Fakler, B. & Dallos, P. Prestin, the Motor Protein of Outer Hair Cells. Audiology and Neurotology **7**, 9–12, doi:10.1159/000046855 (2002). 10.1159/000046855 11914518

[20] Fettiplace R, Kim KX (2014). The Physiology of Mechanoelectrical Transduction Channels in Hearing. Physiological Reviews.

[21] Nam J-H, Peng AW, Ricci AJ (2015). Underestimated Sensitivity of Mammalian Cochlear Hair Cells Due to Splay between Stereociliary Columns. Biophysical Journal.

[22] Eatock RA (2000). Adaptation in Hair Cells. Annual Review of Neuroscience.

[23] Eatock RA, Corey DP, Hudspeth AJ (1987). Adaptation of mechanoelectrical transduction in hair cells of the bullfrog’s sacculus. Journal of Neuroscience.

[24] Fettiplace R, Ricci AJ (2003). Adaptation in auditory hair cells. Curr Opin Neurobiol.

[25] Kennedy HJ, Evans MG, Crawford AC, Fettiplace R (2003). Fast adaptation of mechanoelectrical transducer channels in mammalian cochlear hair cells. Nature neuroscience.

[26] Peng AW, Effertz T, Ricci AJ (2013). Adaptation of mammalian auditory hair cell mechanotransduction is independent of calcium entry. Neuron.

[27] Marcotti W (2016). The acquisition of mechano-electrical transducer current adaptation in auditory hair cells requires myosin VI. The Journal of Physiology.

[28] Ricci AJ, Crawford AC, Fettiplace R (2000). Active Hair Bundle Motion Linked to Fast Transducer Adaptation in Auditory Hair Cells. Journal of Neuroscience.

[29] Rhode, W. S. T. Some observations on two-tone interaction measured with the Mossbauer effect. *Psychophysics and physiology of hearing*, 27–41 (1977).

[30] Ruggero MA, Robles L, Rich NC (1992). Two-tone suppression in the basilar membrane of the cochlea: mechanical basis of auditory-nerve rate suppression. Journal of Neurophysiology.

[31] Sachs MB, Kiang NYS (1968). Two-Tone Inhibition in Auditory-Nerve Fibers. The Journal of the Acoustical Society of America.

[32] Schmiedt RA (1982). Boundaries of two-tone rate suppression of cochlear-nerve activity. Hearing Research.

[33] Delgutte B (1990). Two-tone rate suppression in auditory-nerve fibers: Dependence on suppressor frequency and level. Hearing Research.

[34] Cheatham MA, Dallos P (1989). Two-tone suppression in inner hair cell responses. Hearing Research.

[35] Nuttall AL, Dolan DF (1993). Two-tone suppression of inner hair cell and basilar membrane responses in the guinea pig. The Journal of the Acoustical Society of America.

[36] Rhode WS, Cooper NP (1993). Two-tone suppression and distortion production on the basilar membrane in the hook region of cat and guinea pig cochleae. Hearing Research.

[37] Cooper NP (1996). Two-tone suppression in cochlear mechanics. The Journal of the Acoustical Society of America.

[38] Geisler CD, Nuttall AL (1997). Two-tone suppression of basilar membrane vibrations in the base of the guinea pig cochlea using “low-side” suppressors. The Journal of the Acoustical Society of America.

[39] Rhode WS (2007). Mutual suppression in the 6kHz region of sensitive chinchilla cochleae. The Journal of the Acoustical Society of America.

[40] Geisler CD (1992). Two-tone suppression by a saturating feedback model of the cochlear partition. Hearing Research.

[41] Kanis LJ, de Boer E (1994). Two-tone suppression in a locally active nonlinear model of the cochlea. The Journal of the Acoustical Society of America.

[42] Lim K-M, Steele CR (2002). A three-dimensional nonlinear active cochlear model analyzed by the WKB-numeric method. Hearing Research.

[43] Dong W, Olson ES (2016). Two-Tone Suppression of Simultaneous Electrical and Mechanical Responses in the Cochlea. Biophysical Journal.

[44] Meaud J, Grosh K (2014). Effect of the Attachment of the Tectorial Membrane on Cochlear Micromechanics and Two-Tone Suppression. Biophysical Journal.

[45] Liu Y, Gracewski SM, Nam J-H (2015). Consequences of Location-Dependent Organ of Corti Micro-Mechanics. PLOS ONE.

[46] Nam J-H, Fettiplace R (2012). Optimal Electrical Properties of Outer Hair Cells Ensure Cochlear Amplification. PLOS ONE.

[47] Howard J, Hudspeth AJ (1988). Compliance of the hair bundle associated with gating of mechanoelectrical transduction channels in the Bullfrog’s saccular hair cell. Neuron.

[48] Tinevez J-Y, Jülicher F, Martin P (2007). Unifying the Various Incarnations of Active Hair-Bundle Motility by the Vertebrate Hair Cell. Biophysical Journal.

[49] Dong W, Olson ES (2006). Middle Ear Forward and Reverse Transmission in Gerbil. Journal of Neurophysiology.

[50] Recio A, Rich NC, Narayan SS, Ruggero MA (1998). Basilar-membrane responses to clicks at the base of the chinchilla cochlea. The Journal of the Acoustical Society of America.

[51] de Boer E, Nuttall AL (1997). The mechanical waveform of the basilar membrane. I. Frequency modulations (“glides”) in impulse responses and cross-correlation functions. The Journal of the Acoustical Society of America.

[52] Ruggero MA, Rich NC, Recio A (1996). The effect of intense acoustic stimulation on basilar-membrane vibrations. Auditory Neuroscience.

[53] Meaud J, Lemons C (2015). Nonlinear response to a click in a time-domain model of the mammalian ear. The Journal of the Acoustical Society of America.

[54] Li Y, Grosh K (2016). The Coda of the Transient Response in a Sensitive Cochlea: A Computational Modeling Study. PLOS Computational Biology.

[55] Robles L, Rhode WS, Geisler CD (1976). Transient response of the basilar membrane measured in squirrel monkeys using the Mössbauer effect. The Journal of the Acoustical Society of America.

[56] Ren T, He W, Gillespie PG (2011). Measurement of cochlear power gain in the sensitive gerbil ear. Nature communications.

[57] Rhode WS, Recio A (2001). Multicomponent stimulus interactions observed in basilar-membrane vibration in the basal region of the chinchilla cochlea. The Journal of the Acoustical Society of America.

[58] Ricci AJ, Kennedy HJ, Crawford AC, Fettiplace R (2005). The Transduction Channel Filter in Auditory Hair Cells. Journal of Neuroscience.

[59] Beurg M, Nam J-H, Crawford A, Fettiplace R (2008). The Actions of Calcium on Hair Bundle Mechanics in Mammalian Cochlear Hair Cells. Biophysical Journal.

[60] Peng AW, Gnanasambandam R, Sachs F, Ricci AJ (2016). Adaptation Independent Modulation of Auditory Hair Cell Mechanotransduction Channel Open Probability Implicates a Role for the Lipid Bilayer. J Neurosci.

[61] Kim DO, Molnar CE, Pfeiffer RR (1973). A system of nonlinear differential equations modeling basilar-membrane motion. J Acoust Soc Am.

[62] Allen JB (1977). Two-dimensional cochlear fluid model: new results. J Acoust Soc Am.

[63] Geisler CD (1991). A cochlear model using feedback from motile outer hair cells. Hearing Research.

[64] Yoon, Y. J., Steele, C. R. & Puria, S. Feed-forward and feed-backward amplification model from cochlear cytoarchitecture: an interspecies comparison. *Biophys J* **100**, 1–10, doi:S0006-3495(10)01437-2 [pii], 10.1016/j.bpj.2010.11.039 (2011). 10.1016/j.bpj.2010.11.039 PMC3010833 21190651

[65] Verhulst S, Dau T, Shera CA (2012). Nonlinear time-domain cochlear model for transient stimulation and human otoacoustic emission. The Journal of the Acoustical Society of America.

[66] Liu YW, Neely ST (2010). Distortion product emissions from a cochlear model with nonlinear mechanoelectrical transduction in outer hair cells. J Acoust Soc Am.

[67] Meaud J, Grosh K (2011). Coupling Active Hair Bundle Mechanics, Fast Adaptation, and Somatic Motility in a Cochlear Model. Biophysical Journal.

[68] Stauffer EA, Holt JR (2007). Sensory Transduction and Adaptation in Inner and Outer Hair Cells of the Mouse Auditory System. Journal of neurophysiology.

[69] Géléoc GS, Lennan GW, Richardson GP, Kros CJ (1997). A quantitative comparison of mechanoelectrical transduction in vestibular and auditory hair cells of neonatal mice. Proceedings of the Royal Society B: Biological Sciences.

[70] Johnson SL, Beurg M, Marcotti W, Fettiplace R (2011). Prestin-Driven Cochlear Amplification Is Not Limited by the Outer Hair Cell Membrane Time Constant. Neuron.

[71] Bosher SK, Warren RL (1978). Very low calcium content of cochlear endolymph, an extracellular fluid. Nature.

[72] Wu Y-C, Ricci AJ, Fettiplace R (1999). Two Components of Transducer Adaptation in Auditory Hair Cells. Journal of Neurophysiology.

[73] Corns LF, Johnson SL, Kros CJ, Marcotti W (2014). Calcium entry into stereocilia drives adaptation of the mechanoelectrical transducer current of mammalian cochlear hair cells. Proceedings of the National Academy of Sciences.

[74] Cheung ELM, Corey DP (2006). Ca2+ Changes the Force Sensitivity of the Hair-Cell Transduction Channel. Biophysical Journal.

[75] Assad JA, Corey DP (1992). An active motor model for adaptation by vertebrate hair cells. Journal of Neuroscience.

[76] Fettiplace R, Ricci AJ, Hackney CM (2001). Clues to the cochlear amplifier from the turtle ear. Trends in Neurosciences.

[77] Cooper NP, Rhode WS (1996). Fast travelling waves, slow travelling waves and their interactions in experimental studies of apical cochlear mechanics (vol 2, pg 207, 1996). Auditory Neuroscience.

