# Supplementary material for: Probing hair cell’s mechano-transduction using two-tone suppression measurements
Source: Sci Rep. 2019 Mar 15;9:4626. doi: 10.1038/s41598-019-41112-5 (PMC6420497; doi:10.1038/s41598-019-41112-5)
Supplement: Supplementary file 1 — Supporting Materials [file 41598_2019_41112_MOESM1_ESM.docx]

**Title**: Probing hair cell’s mechano-transduction using two-tone suppression measurements

**Authors**: Wenxiao Zhou^1,*^, and Jong-Hoon Nam^1, 2^

Affiliation:

^1^Department of Mechanical Engineering, University of Rochester, Rochester, NY 14627, USA

^2^Department of Biomedical Engineering, University of Rochester, Rochester, NY 14627, USA

# Supporting Materials

**Finite element method for the fluid domain:**

The geometry of the fluid domain is shown in Fig.1A, and finite element method is applied to the Laplace equation (Eqn.1). Adaptive mesh is generated with triangle elements using customized Matlab mesh generating function. At both interacting surfaces, the length of one element side is set to be 10um, so that it is compatible with the space between adjacent sections of the OCC model. Because fluid pressure gradient is more important around the tissue, coarser mesh is allowed away from the organ of Corti to save computational cost without sacrificing accuracy of the results.

For 2D Laplace equation, the weak formulation of the weighted residual of the differential equation can be written as:

$I=-\int_{\Omega} \left( \frac{\partial w}{\partial x}\frac{\partial p}{\partial z}+\frac{\partial p}{\partial x}\frac{\partial w}{\partial z} \right)d\Omega+\int_{\Gamma} w\frac{\partial p}{\partial n}d\Gamma$ (S1)

Where $\omega$ is the weight function and $p$ is the unknown pressure field. The first volume integration becomes a matrix term analogous to stiffness matrix in mechanical problem, while the second line integration denotes the kinematic boundary condition given by the interacting surfaces.

For a triangle element with nodes at (*x*_1_, *z*_1_), (*x*_2_, *z*_2_) and (*x*_3_, *z*_3_), we have linear shape functions that satisfy the conditions:

$H_{i}\left( x_{i},z_{j} \right)=\delta_{ij}, \left( i,j=1,2,3 \right)$ (S2)

$\sum_{i=1}^{3} H_{i}=1$ (S3)

Where $\delta_{ij}$ is the Kronecker delta.

The element matrix for the fluid domain is given as:

$\mathbf{A}_{pp}^{e}=\int_{\Omega e} \left( \frac{\partial\mathbf{H}^{T}}{\partial x}\frac{\partial\mathbf{H}}{\partial x}+\frac{\partial\mathbf{H}^{T}}{\partial z}\frac{\partial\mathbf{H}}{\partial z} \right)d\Omega$ (S4)

The boundary integral along an element length at the interacting surface becomes:

$\mathbf{A}_{pa}^{e}{\ddot{\mathbf{x}}}^{e}=\pm\frac{l^{e}}{2}\rho\left[ \begin{matrix} \ddot{x}_{1}^{e} \\ \ddot{x}_{2}^{e} \end{matrix} \right]$ (S5)

Where $l^{e}$ is the boundary length of the element. $\ddot{x}_{1}^{e}$ and $\ddot{x}_{2}^{e}$ are the accelerations from the two nodes on the boundary. The sign depends on whether it’s on the basilar membrane side or the tectorial membrane side.

**Numerical methods to solve the governing equation in the time domain:**

The discretized governing equation (Eq. 18) was integrated over time using the Newmark’s method and reorganized in the vector-matrix form:

$\left( \mathbf{K}^{\mathbf{'}}+\mathbf{K} \right)\mathbf{x}^{m+1}-\left( {\mathbf{f}_{EFF}}^{m+1}+{\mathbf{f}_{\mathrm{MET}}}^{m+1}+{\mathbf{f}_{\mathrm{OHC}}}^{m+1} \right)=\mathbf{K}^{\mathbf{'}}\mathbf{x}^{m}+\mathbf{C}^{\mathbf{'}}{\dot{\mathbf{x}}}^{m}+\mathbf{M'}{\ddot{\mathbf{x}}}^{m}$, (S10)

where

$\mathbf{K}^{\mathbf{'}}=\frac{\mathbf{M}_{EFF}}{a_{2}{\Delta t}^{2}}+\frac{a_{1}}{a_{2}\Delta t}\mathbf{C}$, (S11)

$\mathbf{C}^{\mathbf{'}}=\frac{\mathbf{M}_{EFF}}{a_{2}\Delta t}+\left( \frac{a_{1}}{a_{2}}-1 \right)\mathbf{C}$, (S12)

$\mathbf{M}^{\mathbf{'}}\mathbf{=}\left( \frac{1}{2a_{2}}-1 \right)\mathbf{M}_{EFF}+\Delta t\left( \frac{a_{1}}{2a_{2}}-1 \right)\mathbf{C}$. (S13)

$a_{1}=$ 0.5005 and $a_{2}=$ 0.3333 are relaxation factors. $\Delta t$ is the time step size with default value of $4 \mu s$. The displacement of the structures at the (m+1)^th^ time step was solved in Eq. S10. The velocity and acceleration at the (m+1)^th^ time step are:

${\dot{\mathbf{x}}}^{m+1}=\frac{a_{1}}{a_{2}\Delta t}\left( \mathbf{x}^{m+1}-\mathbf{x}^{m} \right)+\left( 1-\frac{a_{1}}{a_{2}} \right){\dot{\mathbf{x}}}^{m}+\Delta t\left( 1-\frac{a_{1}}{2a_{2}} \right){\ddot{\mathbf{x}}}^{m}$, (S14)

${\ddot{\mathbf{x}}}^{m+1}=\frac{1}{a_{2}{\Delta t}^{2}}\left( \mathbf{x}^{m+1}-\mathbf{x}^{m} \right)-\frac{1}{a_{2}\Delta t}{\dot{\mathbf{x}}}^{m}-\left( \frac{1}{2a_{2}}-1 \right){\ddot{\mathbf{x}}}^{m}$. (S15)

**Table S1. Mechanical parameters of OCC**

| Component | Parameters | *x* = 2 mm | *x* = 10 mm | Unit | Ref |
| --- | --- | --- | --- | --- | --- |
| Basilar membrane | Width (arcuate, pectinate)  Thickness (arcuate, pectinate)  YM (radial, longitudinal) | 53, 107  0.6, 3  1000, 0.4 | 93,187  0.14,0.7  1000,0.1 | μm  μm  MPa | ^1-3^ |
| OHC | Diameter, Length  YM | 9, 20  0.09 | 9, 50  0.09 | μm  MPa | ^4,5^ |
| OHC  Hair bundle | Height, Width  Stiffness | 2, 8  40 | 6,8  40 | μm  mN/m | ^6-9^ |
| Pillar cell | Diameter  YM | 8  10 | 4  10 | μm  MPa | ^10^ |
| Deiters cell | Diameter (body, phalange)  YM (body, phalange) | 10, 1.5  0.5, 3 | 10, 1  0.5, 3 | μm  MPa | ^10^ |
| Reticular lamina | Thickness (pillar cell, OHC)  YM (radial, longitudinal) | 5, 2  10, 0.2 | 5, 1  2, 0.05 | μm  MPa | - |
| Tectorial membrane | Width (body, root)  Thickness (body, root)  Radial YM (body, root)  Longitudinal YM | 53, 27  30, 15  0.2, 0.8  0.002 | 140, 70  50, 25  0.01, 0.04  0.002 | μm  μm  MPa  MPa | ^2,11-14^ |

**Table S2. OHC parameters**

| Component | Parameter | x=2 mm | x=10 mm | Unit | Ref |
| --- | --- | --- | --- | --- | --- |
| MET channel | Geometric gain, γ  Gating spring stiffness, *k_G_*  Gating swing, *b*  Number of channels, *N*  Activation rate constant, *A*_0_  Adaptation rate constant, *k_A_*  Resting open probability, *p_o,rest_* | 0.25  6  0.13  75  100  15  0.45 | 0.1  6  0.13  60  10  15  0.45 | -  mN/m  nm  -  ms^-1^  nm/ms/pN  - | ^6,15^  ^6,15^  ^6,15^  ^6,15^  ^6,15^  ^16^  ^6,15^ |
| Stereocilia | Max conductance, *G_S,max_*  Capacitance, *C_S_*  Max reactive force, *f_MET,max_* | 90  2.6  100 | 27  9  12 | mV  nS  pN | ^17^ |
| Basolateral membrane | Equilibrium potential, *E_K_*  Resting potential, *V_m,rest_*  Conductance, *G_m_*  Capacitance, *C_m_*  Active gain, *α_OHC_* | 75  -53  230  4.3  0.1 | 75  -37  39  15  0.1 | mV  mV  nS  pF  nN/mV | ^17^ |

**Cochlear responses at the apical location show qualitative difference compared to the basal location**

Impulse and pure tone responses at the apical location (*x* = 8 mm) are shown below. Compared to responses at the basal location, the impulse response exhibits fewer cycles of vibration and quick decay in amplitude. IF can only be obtained for about 1 ms and still shows an increasing trend. For the traveling wave envelope seen in 1 kHz pure tone response, the 3 dB spatial width is about 1.5 mm and the half wavelength is about 1 mm. The sharpness of the pure tone response decreases significantly compared to the basal location and the compressive nonlinearity is much weaker. With 70 dB increase in the input level, the basilar membrane displacement gain changes by 7 dB and the peak location moves slightly towards the base. The lack of nonlinearity is also shown in the peak amplitude in impulse response and pure tone response amplitude as a function of the input level. In both cases, at intermediate and high levels (>85 dB re. StP), the growth rates are above 0.8 dB/dB on average.


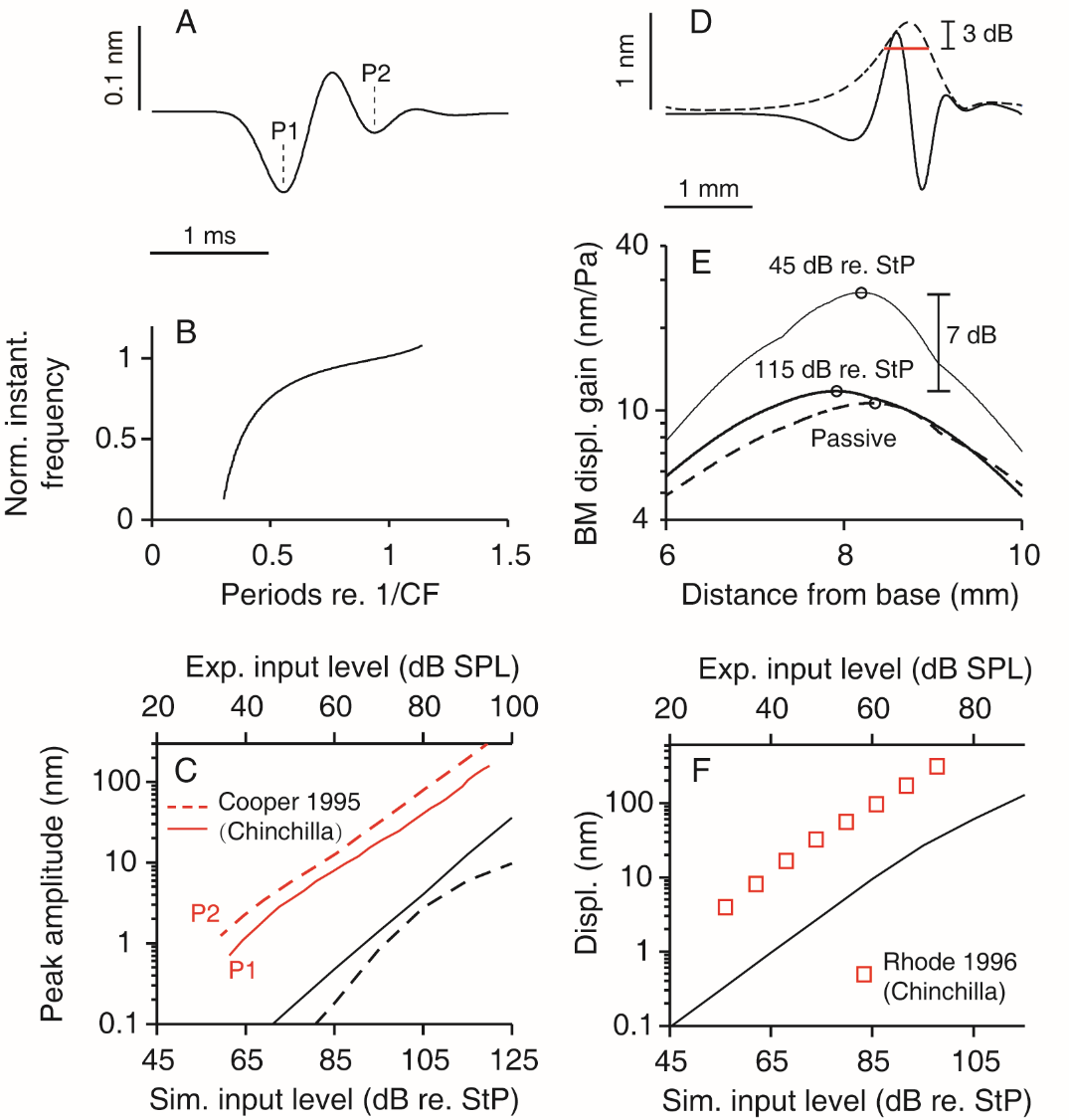


**Figure S1**. **Impulse and pure tone responses at 8mm location**

(**A**) and (**D**) Basilar membrane responses to a click and a 1 kHz pure tone. (**B**) Instantaneous frequency (IF) of the temporal response normalized with CF as a function of time. (**C**) basilar membrane peak amplitude as a function of input level from simulation (black) and experimental data (red). (**E**) Traveling wave envelopes for 1 kHz pure tone at 45, 115 dB re. StP and passive case (w/o OHC active forces). (**F**) Basilar membrane displacement at the best frequency location as a function of the input level. Solid line and circles represent simulation results and experiment data, respectively.

**Expansion of saturating region with increase of input level**

As vibration amplitude increases, MET current starts to saturate and limits the energy produce by the outer hair cell. Fig.S1 shows the region of MET saturation (where change of MET current exceeds 0.9 of the maximum value) at different input levels. At intermediate levels, saturation concentrates at the peak location. As input level increases, distribution of saturation spread towards both basal and apical locations.


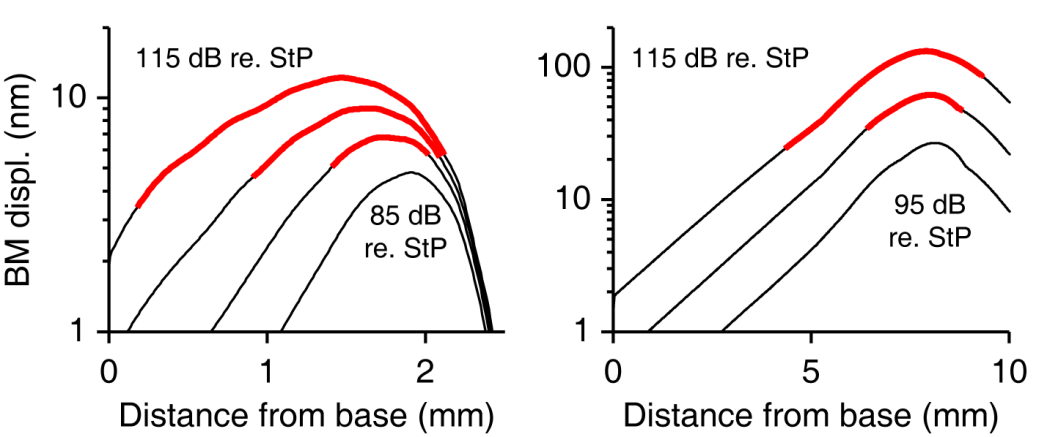


**Figure S2: Distribution of saturation region as input level increases.**

Red curves represent the saturation region. Responses are shown for 17 kHz (left) and 1 kHz (right) pure tone with 85~115 dB re. StP and 95~115 dB re. StP, respectively. Input increment is 10 dB.

2TS temporal patterns are sensitive to input frequency ratio and resting open probability.

Geisler and Nuttall reported that temporal pattern of probe response can follow suppressor displacement faithfully until 2 kHz. It implies the frequency ratio needs to be lower than a certain level for the response to fully recover from maximum suppression. For our simulation, strong phasic suppression can only be observed with suppressors less than 1.5 kHz for the 17 kHz probe tone. For higher frequencies, the tonic suppression dominates and fewer cycles of probe tone vibration are there within one cycle of suppressor response (Fig. S3). This phenomenon might be caused by the different time constants associated with probe tone suppression and recovery. Since it takes some time for the amplification to increase the probe response, there may not be enough time before the suppressor displacement increases and suppresses the probe response again. Therefore, it is difficult to determine the maximum suppression phase with suppressors > 1.7 kHz.


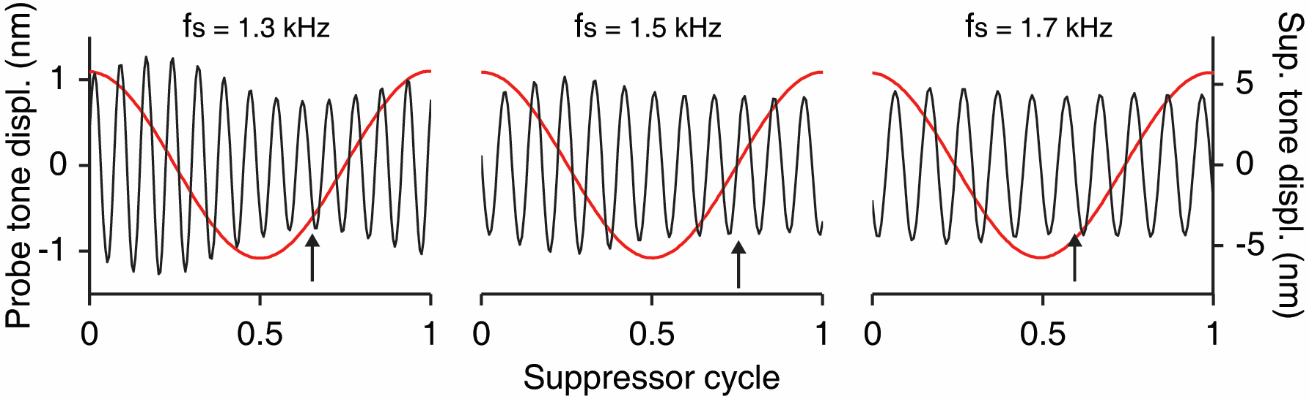


**Figure S3: Temporal patterns of 2TS with suppressor frequency > 1 kHz.**

Black and red curves represent probe tone and suppressor tone displacement within one cycle of suppressor tone vibration. Arrows indicate maximum suppression

Bias of open probability can affect the timing of maximum suppression relative to the maximum displacement of suppressor tone towards either scala vestibuli or scala tympani. For open probability less than 0.4, displacement of suppressor towards scala tympani causes greater saturation of MET current of the probe tone, therefore maximum suppression follows largest displacement towards scala tympani. If open probability is biased above 0.5, then the trend reverses. Fig.S3 shows the two distinct situations. Since MET sensitivity is set to be the highest when open probability is equal to 0.4, changing resting open probability to 0.6 also reduces overall probe tone amplitude.


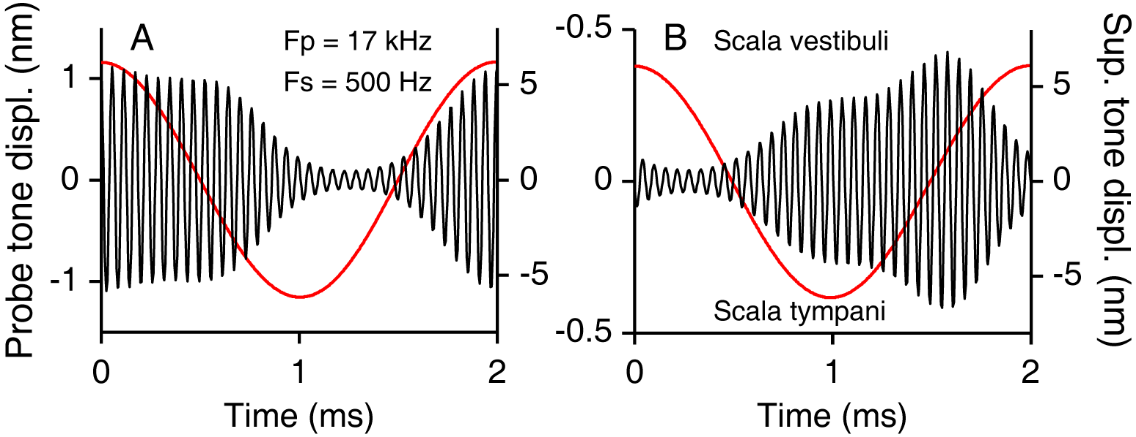


**Figure S4: Bias of resting open probability determines polarity of suppressor displacement near maximum suppression**

(**A**) Temporal pattern of probe tone (17 kHz) and suppressor tone (500 Hz) with resting open probability set to be 0.4. (**B**) Same plot with resting open probability at 0.6.

**Effect of adaptation extent on phase change**

Adaptation extent (percentage of MET current decay relative to its maximal value) affects filtering on mechanotransduction. Here we show phase shift of MET with a 1 kHz suppressor tone at different extents. Phase shift increases monotonically as extent of adaptation increases. When there is no adaptation (0%, MET current will not decay), phase shift is zero and when there is complete adaptation (100%, MET current totally disappears given enough time), phase shift is maximal. For the simulation in Fig. 9, we used complete adaptation with cut-off frequency at ~4 kHz, which resulted in phase shifts at the higher end. For more realistic situation, we expect the phase shift to be lower especially for low frequency suppressors.


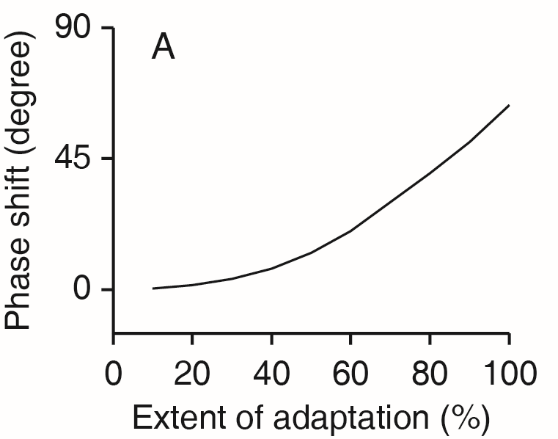


**Figure S5: Phase shift as a function of adaptation extent and cut-off frequency**

(A) Mechanotransduction phase change as a function of adaptation extent. Adaptation cut-off frequency is 4 kHz and simulation is at 17 kHz best frequency location for a 1 kHz suppressor tone.

**References**

1 Plassmann, W., Peetz, W. & Schmidt, M. 82-102 (1987).

2 Schweitzer, L., Lutz, C., Hobbs, M. & Weaver, S. P. Anatomical correlates of the passive properties underlying the developmental shift in the frequency map of the mammalian cochlea. *Hearing Research* **97**, 84 - 94 (1996).

3 Edge, R. M. *et al.* Morphology of the unfixed cochlea. *Hearing Research* **124**, 1 - 16 (1998).

4 Iwasa, K. H. & Adachi, M. Force generation in the outer hair cell of the cochlea. *Biophysical Journal* **73**, 546 - 555 (1997).

5 He, D. Z. Z., Evans, B. N. & Dallos, P. First appearance and development of electromotility in neonatal gerbil outer hair cells. *Hearing Research* **78**, 77 - 90 (1994).

6 Beurg, M., Nam, J.-H., Crawford, A. & Fettiplace, R. The Actions of Calcium on Hair Bundle Mechanics in Mammalian Cochlear Hair Cells. *Biophysical Journal* **94**, 2639-2653 (2008).

7 Roth, B. & Bruns, V. Postnatal development of the rat organ of Corti. *Anatomy and Embryology* **185**, 571-581 (1992).

8 Lim, D. J. Functional structure of the organ of Corti: a review. *Hearing Research* **22**, 117 - 146 (1986).

9 Strelioff, D. & Flock, Å. Stiffness of sensory-cell hair bundles in the isolated guinea pig cochlea. *Hearing Research* **15**, 19 - 28 (1984).

10 Zetes, D. E., Tolomeo, J. A. & Holley, M. C. Structure and Mechanics of Supporting Cells in the Guinea Pig Organ of Corti. *PLOS ONE* **7**, 1-9 (2012).

11 Edge, R. M. *et al.* Morphology of the unfixed cochlea. *Hearing Research* **124**, 1-16, doi:<https://doi.org/10.1016/S0378-5955(98)00090-2> (1998).

12 Gueta, R., Barlam, D., Shneck, R. Z. & Rousso, I. Measurement of the mechanical properties of isolated tectorial membrane using atomic force microscopy. *Proceedings of the National Academy of Sciences* **103**, 14790-14795 (2006).

13 Gu, J. W., Hemmert, W., Freeman, D. M. & Aranyosi, A. J. Frequency-Dependent Shear Impedance of the Tectorial Membrane. *Biophysical Journal* **95**, 2529-2538 (2008).

14 Richter, C.-P., Emadi, G., Getnick, G., Quesnel, A. & Dallos, P. Tectorial Membrane Stiffness Gradients. *Biophysical Journal* **93**, 2265-2276 (2007).

15 Nam, J.-H. & Fettiplace, R. Theoretical Conditions for High-Frequency Hair Bundle Oscillations in Auditory Hair Cells. *Biophysical Journal* **95**, 4948 - 4962 (2008).

16 Kennedy, H. J., Evans, M. G., Crawford, A. C. & Fettiplace, R. Fast adaptation of mechanoelectrical transducer channels in mammalian cochlear hair cells. *Nature neuroscience* **6**, 832-836 (2003).

17 Johnson, Stuart L., Beurg, M., Marcotti, W. & Fettiplace, R. Prestin-Driven Cochlear Amplification Is Not Limited by the Outer Hair Cell Membrane Time Constant. *Neuron* **70**, 1143 - 1154 (2011).
